# Trends in the Research Into Immune Checkpoint Blockade by Anti-PD1/PDL1 Antibodies in Cancer Immunotherapy: A Bibliometric Study

**DOI:** 10.3389/fphar.2021.670900

**Published:** 2021-08-17

**Authors:** Yiting Sun, Liqing Jiang, Ti Wen, Xiaoyu Guo, Xinye Shao, Hui Qu, Xi Chen, Yujia Song, Fang Wang, Xiujuan Qu, Zhi Li

**Affiliations:** ^1^ Department of Medical Oncology, The First Hospital of China Medical University, Shenyang, China; ^2^ Key Laboratory of Anticancer Drugs and Biotherapy of Liaoning Province, The First Hospital of China Medical University, Shenyang, China; ^3^ Liaoning Province Clinical Research Center for Cancer, The First Hospital of China Medical University, Shenyang, China; ^4^ Key Laboratory of Precision Diagnosis and Treatment of Gastrointestinal Tumors, Ministry of Education, The First Hospital of China Medical University, Shenyang, China

**Keywords:** PD1/PDL1, immune checkpoint blockade, bibliometrics, randomized clinical trials, meta-analysis

## Abstract

The programmed death receptor 1 (PD1) and its ligand programmed death receptor ligand 1 (PDL1) are the most widely used immune checkpoints in cancer immunotherapy. The related literature shows the explosive growth trends due to the promising outcomes of tumor regression. The present study aimed to provide a comprehensive bibliometric analysis of the literature on anti-PD1/PDL1 from three perspectives including molecular mechanisms, randomized clinical trials (RCT), and meta-analysis, thus producing a knowledge map reflecting the status of the research, its historical evolution, and developmental trends in related research from 2000 to 2020. We included 11,971, 191, and 335 documents from the Web of Science Core Collection database, respectively, and adopted various bibliometric methods and techniques thereto. The study revealed the major research themes and emergent hotspots based on literature and citation data and outlined the top contributors in terms of journals and countries. The co-occurrence overlay of keywords and terms pertaining to the PD1/PDL1 molecule reflected the progress from the discovery of the PD1/PDL1 molecule to the clinical application of anti-PD1/PDL1. Immune-related adverse events (irAEs) formed a unique cluster in the term co-occurrence analysis of meta-analysis. The historical direct citation network of RCT indicated the development and transformation of cancers and therapy strategies. irAEs and the strategies of combination therapy might become a future focus of research in this cognate area. In summary, the bibliometric study provides a general overview of the landscape on anti-PD1/PDL1 research, allowing researchers to identify the potential opportunities and challenges therein.

## Introduction

Cancer is a public health problem and a major cause of mortality with significant repercussions on individual patients and societies. The strategies of cancer treatment have undergone a unique evolution (Mellman et al., 2011). The development of cancer immunotherapy has changed our understanding of cancer biology and the manners of treatment (Pardoll, 2012; Osipov et al., 2019). It can enhance antitumor responses by regulating the host immune system compared with traditional methods. Cancer immunotherapy is a rapidly growing field, and many immunotherapeutic agents including vaccine-based therapies, oncolytic viruses and T cell directed therapies have been approved in various cancers (Khalil et al., 2016). Among which, the immune checkpoint blockade (ICB) has achieved great success due to its promising outcomes with regard to tumor regression (Liu et al., 2020).

PD1 and its ligand PDL1 were the most widely used immune checkpoints in clinical practice. PD1 is a type-1 transmembrane protein which was first discovered in 1992 (Ishida et al., 1992). It is remarkably expressed on the surface of many effector immune cells including T cells, B cells, dendritic cells, monocytes and tumor-infiltrating lymphocytes (TILs). PDL1, a member of the B7 family of co-stimulatory/co-inhibitory molecules of antigen presentation, was independently discovered by two research teams in 1999 and 2000 (Dong et al., 1999; Freeman et al., 2000). It is expressed in cancer cells and many antigen-presenting cells (APCs) (Zuazo et al., 2017). Inflammatory cytokines are produced when T cells recognize the antigen expressed by major histocompatibility complex (MHC) on target cells. The action and blocking mechanisms of PD1 and PDL1 are as follows. The activation of T cells contributes to the expression of PDL1 on the surface of cancer cells and PD1 on various immune cells. The combination of PD1 and PDL1 molecule causes T cell dysfunction and exhaustion as well as immune tolerance within the tumor microenvironment (Alsaab et al., 2017). The blockade of the PD1/PDL1 axis by anti-PDL1 could affect antitumor immune response and suppress tumor growth since it was first demonstrated in the PDL1+ mouse model in 2002, providing attractive targets for cancer immunotherapy (Dong et al., 2002). Blockade of PD1 or PDL1 could recover anti-tumor immunity mediated by T cell because of preventing the interaction between molecules (Kwok et al., 2016). The proliferation and effector functions of T cells could also be inhibited by the combination of PD1 and its another ligand programmed death receptor ligand 2 (PDL2) (Chang et al., 2018). Due to the weaker binding affinity of PD1 and PDL2 and the restricted expression of PDL2, its application in cancer immunotherapy is limited (Alsaab et al., 2017). Furthermore, CD80-Fc could bind with PDL1 to prevent PD1-PDL1-mediated suppression and facilitate T cell activation by co-stimulating through CD28 (Haile et al., 2013). These should also be taken into consideration during PD1/PDL1 blockade.

With the wide application of anti-PD1/PDL1 therapy over recent years, researchers found that a sizeable proportion of patients do not show clinical responses or develop acquired resistance after initial responses. Therefore, the scientists focus on the determinants driving the response, the mechanisms of resistance, potential biomarkers for clinical benefit, and the strategies of combined therapy to improve anti-PD1/PDL1 efficacy (Hack et al., 2020; Jafarzadeh et al., 2020; Lei et al., 2020). Besides, irAEs have been reported in the treatment of anti-PD1/PDL1 including rashes, pneumonitis, colitis, hepatitis, myocarditis, hypophysitis, and so forth. Although the incidences thereof are not high, these might affect patient quality of life and can even be fatal (Dupont et al., 2020; Joseph et al., 2020). The widespread concern about anti-PD1/PDL1 in cancer treatment advances the processing of related research, and the body of literature is growing rapidly. Hence, the programmatic and instructive review is necessary to disentangle the results and indicate directions.

The traditional review usually reflects the recent progress in a certain aspect of a topic instead of the overall landscape of the discipline. Bibliometrics is a measurable informatic method that analyses the knowledge structure to obtain quantifiable data and to address the above limitation (Guo et al., 2020; McElroy and Allen, 2020; Wang et al., 2020). Although there have been several bibliometric studies related to PD1 or PDL1, they usually only focused on some specific aspects such as documents on PD1/PDL1 molecule, anti-PD1/PDL1 therapy in China or for a single kind of cancer (Zhao et al., 2018; Baş and Şenel, 2019; Gao et al., 2019; Ahn and Hwang, 2020; Li et al., 2021). Considering that the application of anti-PD1/PDL1 in various cancers is still in exploration, and researchers devote to explore more mechanism of PD1/PDL1 molecule to improve efficacy of immunotherapy, it is necessary to conduct an updated and more comprehensive bibliometric study. The current study focused on the publications on the PD1/PDL1 molecule and RCT as well as meta-analysis of anti-PD1/PDL1 therapy to provide a holistic view of related research to benefit researchers and patients for the first time. We found that the documents of these three aspects could represent the determinants driving the response, clinical application, and adverse effects of anti-PD1/PDL1, respectively. Combining the three perspectives of the results would give us a new and overall understanding.

The purposes and benefits of this study include the following several points. The study provided the complete overview of academic structure in PD1/PDL1 research based on literature and citation data to benefit researchers and patients. Firstly, we present the current status of related research by summarizing trends of production, top contributors, major research themes and highly cited documents. Besides, the historical evolution of the field was outlined by bibliographic coupling overlay and historical direct citation network. Finally, keyword and term co-occurrence overlays and references with citation bursts were analyzed to reflect the focus of future studies. This information should be helpful to readers, including those without deep previous knowledge of the topic, in gleaning a general overview of the landscape, including the historical evolution and the future focus of the field. The information could also be used to identify potentially promising research directions, possible collaboration partners, and relevant publications.

## Materials and Methods

### Data Source and Search Strategies

A comprehensive search was performed online using the Citation Index Expanded (SCI-E) database from Web of Science Core Collection (WoSCC) through the Library of China Medical University on May 22, 2020. The database was selected to conduct the bibliometric study because it provided plenty of bibliometric indicators including publication and citation data. The study included three datasets, including the documents of the PD1/PDL1 molecule (dataset A), RCT (dataset B) and meta-analysis (dataset C) of anti-PD1/PDL1. The search terms were PD1/PDL1, various anti-PD1/PDL1 agents, cancer, meta-analysis and their synonyms. Besides, various writing formats and search rules of database were considered to cover as many results related in the process of designing the search strategies. The detailed search strategies were presented in Supplementary Material S1.

### Data Filtration

**FIGURE 1:**
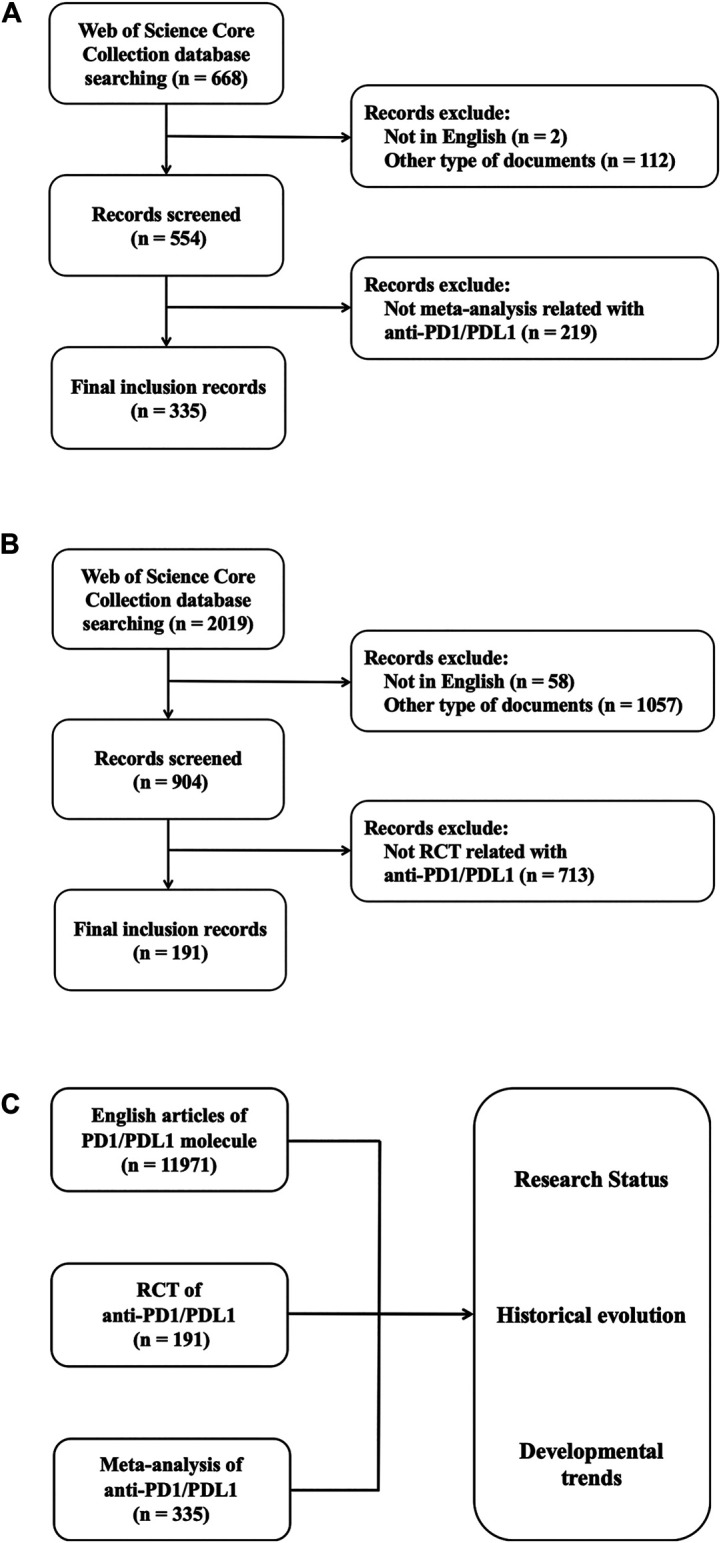
Data filtration processing and results. Flowchart of inclusion and exclusion criteria for meta-analysis of anti-PD1/PDL1 **(A)** and RCT of anti-PD1/PDL1 **(B)**. **(C)** Schematic diagram of main research contents.

According to the search strategy, 22,659 records were found for dataset A. The publications were limited to English articles and we included 11,971 documents eventually. 2,019 records were searched for dataset B at first. We excluded the records published not in English or with the publication type of review, meeting abstract, editorial material and correction, and 904 documents were exported to screen further. In dataset C, 554 in 668 documents were exported because the records published in non-English languages or in the type of meeting abstract and correction were eliminated. After the initial search, manual filtration was performed by viewing the contents of documents to screen the documents with the type of RCT (including their secondary analysis and study designs) and meta-analysis for dataset B and C, respectively. Then we further judged whether there was a clear correlation with anti-PD1/PDL1. 713 and 219 unrelated records were excluded in dataset B and C, respectively, most of which focused on PD1/PDL1 expression or did not conform with concerned document types. The meta-analysis with the publication type of review was included in dataset C. Finally, there were 191 and 335 results identified as valid after comparing the outcomes of filtration made by at least two authors independently in dataset B and C. The filtering process is shown in Figure 1. The information of screened results for 3 datasets were exported in the formats of BIB, UTF-8 encoded TXT and TXT to conduct the bibliometric analysis.

### Data Analysis and Visualization

We applied several methods to represent the bibliographic data. The number of productions and citations (including total citations and average citations per paper) were considered as the most important bibliometric indicators because they represented productivity and influence, respectively (Svensson, 2010; Ding et al., 2016). Besides, we included other common measures such as international collaboration of countries, top contributors in terms of authors, institutions, countries and journals, as well as historical direct citation network (Mulet-Forteza et al., 2019a; Mulet-Forteza et al., 2019b; Merigó et al., 2019). Local citation score (LCS) represents the number of times that an article has been referenced in the current dataset, therefore, a high LCS indicated the importance of the article in the field. Besides, the global citation score (GCS) was also been calculated, which is the number of times the article has been cited by all documents in the entire WoSCC database, and it covers the influence of the article on other areas (Wang et al., 2021). The citation thresholds were set to identify the number of influential documents and we also concerned the citations per year since their publication to define the references with citation bursts (Mulet-Forteza et al., 2019a; Merigó et al., 2019).

The different formats of the download files were imported into the R Studio, VOSviewer, and CiteSpace for analysis. The R package bibliometrix was taken to get the basic information of datasets including annual scientific production and percentage growth rate, the production and citations as well as international collaboration of countries, highly cited documents, most relevant journals and the historical direct citation network (Aria and Cuccurullo, 2017). VOSviewer 1.6.15 was used to perform the cluster analysis and visualize the network maps of the keywords, terms, authors, institutions and co-cited references (van Eck and Waltman, 2010; Sinkovics et al., 2016). In the network maps, the nodes represented elements and the size of the nodes reflected the frequency. The links between nodes represented relationships such as co-occurrence, coupling or co-citation. The co-occurrence of the keywords and terms identified the core scientific knowledge in the research field and indicated the correlations between research topics (Su and Lee, 2010). The documents with a coupling relationship shared cited references, measuring the relevance of their research. Conversely, co-citation indicated the similarity between documents when they were cited by the same records (Mulet-Forteza et al., 2019b). The color of nodes and lines represented different clusters or years. The names of the different nodes represented are indicated in Supplementary Table S1 for every figure. Furthermore, CiteSpace 5.7.R1 (64-bit) was used to analysis the references with strongest citation bursts (Chen, 2006). The impact factors (IF) of journals were obtained from the 2019 Journal Citation Reports (JCR) (Clarivate Analytics, Philadelphia, United States). The analysis represented in tables and figures was made using a full counting system in this study, except for term co-occurrence analysis which was conducted by binary counting (van Eck and Waltman, 2010).

## Results

### The Current Status of PD1/PDL1 and anti-PD1/PDL1 Research

**FIGURE 2:**
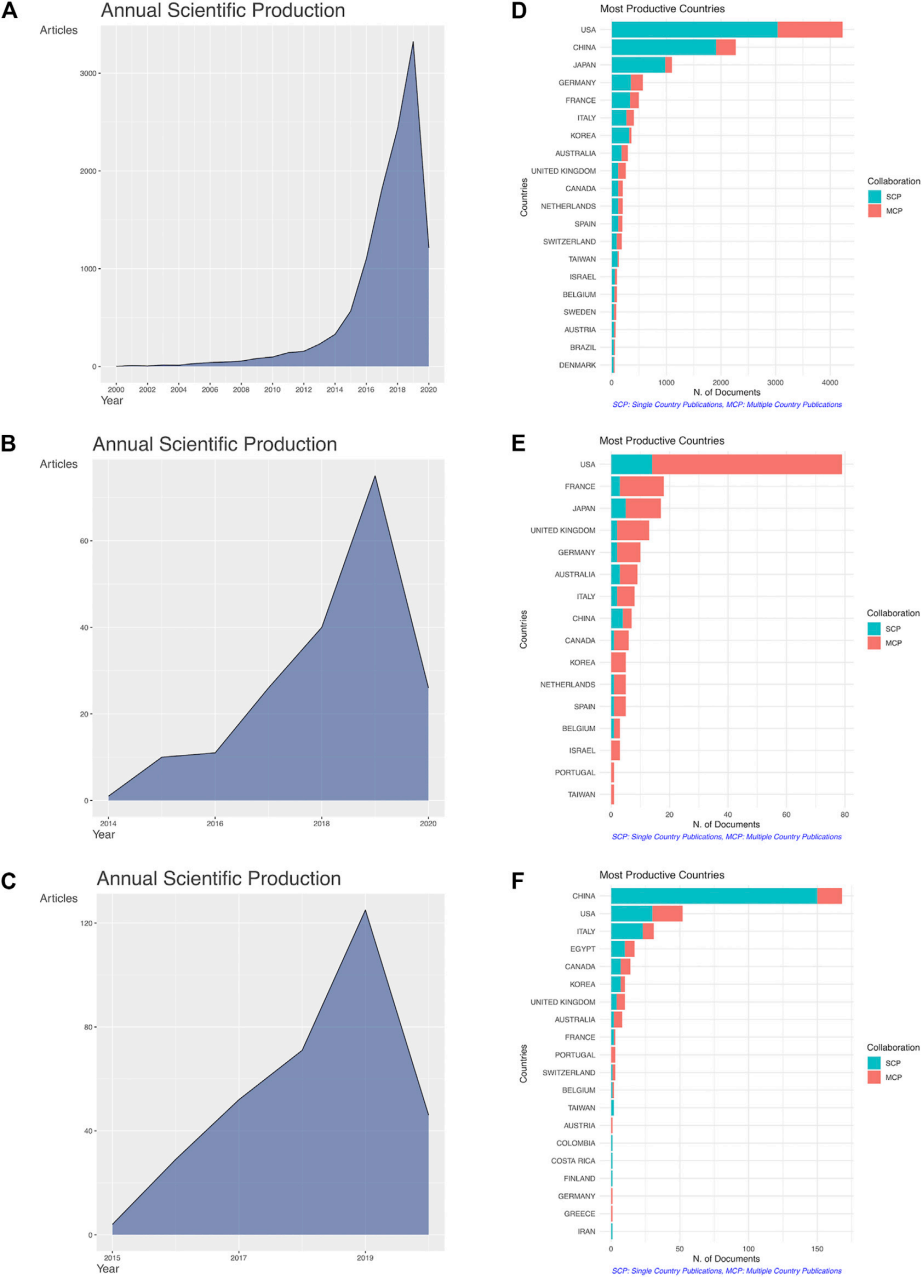
The growth trend in publication number and the international cooperation of countries. Annual scientific production of the articles on the PD1/PDL1 molecule **(A)**, RCT of anti-PD1/PDL1 **(B)** and meta-analysis of anti-PD1/PDL1 **(C)**. Top 20 productive countries and their international collaboration on the PD1/PDL1 molecule **(D)**, RCT of anti-PD1/PDL1 **(E)** and meta-analysis of anti-PD1/PDL1 **(F)**. The blue and red lines represented single country publication (SCP) and multiple countries publication (MCP).

The main information pertaining to the collected bibliometric data is summarized in Table 1. The literature search on the PD1/PDL1 molecule resulted in 11,971 documents from 1,226 sources (journals, books, *etc*.). The growth trend in publication number of PD1/PDL1 molecule was the most obvious, and the field is still in a phase of rapid ascent (Figures 2A-C). Since 2016, 9,888 articles have been published, accounting for 82.60% of the publications in the field. Considering that we limited documents to those marked as having the publication type “article”, the number of items with a focus on the PD1/PDL1 molecule is much greater (Figure 2D). The search of RCT and meta-analysis of anti-PD1/PDL1 returned 191 and 335 documents received an average number of citations per paper of 263.20 and 13.88, respectively. RCT is considered as the gold standard when used to evaluate the safety and efficacy of drugs and it usually requires cooperation between multiple countries and organizations. In the 191 RCT documents on anti-PD1/PDL1, the United States was the most productive country, publishing 79 items (41.36% of the total) including 65 multiple-country publications (Figure 2E). Besides, France (18), Japan (17), the United Kingdom (13), and Germany (10) made significant contributions. Among the 335 meta-analysis documents of anti-PD1/PDL1 (Figure 2F), a large number of publications could be attributed to authors in China (168, 50.20% of the total), United States (52), and Italy (31) (Table 2).

**TABLE 1:** Summary of the main information of collected bibliometric data about three datasets.

	PD1/PDL1 molecule	RCT of anti-PD1/PDL1	Meta-analysis of anti-PD1/PDL1
Documents	11,971	191	335
Timespan	2000 : 2020	2014 : 2020	2015 : 2020
Sources	1,226	45	105
Annual Percentage Growth Rate	37.77	72.12	62.98
Average citations per documents	34.27	263.20	13.88
References	185,462	3,057	6,048
Author’s Keywords	12,134	249	479
Keywords Plus	10,745	413	547

**TABLE 2:** Top 10 productive countries and their total and average citations in three datasets.

PD1/PDL1 molecule				RCT of anti-PD1/PDL1				Meta-analysis of anti-PD1/PDL1			
Country	Records	Total citations	Average citations	Country	Records	Total citations	Average citations	Country	Records	Total citations	Average citations
USA	4,226	254,907	60.32	USA	79	34,771	440.14	CHINA	168	1,032	6.14
CHINA	2,270	23,847	10.51	FRANCE	18	6,940	385.56	USA	52	1734	33.35
JAPAN	1,102	22,537	20.45	JAPAN	17	715	42.06	ITALY	31	468	15.10
GERMANY	569	13,250	23.29	UNITED KINGDOM	13	4,578	352.15	EGYPT	17	405	23.82
FRANCE	495	21,303	43.04	GERMANY	10	882	88.20	CANADA	14	100	7.14
ITALY	405	5,615	13.86	AUSTRALIA	9	413	45.89	KOREA	10	67	6.70
KOREA	361	5,077	14.06	ITALY	8	176	22.00	UNITED KINGDOM	10	98	9.80
AUSTRALIA	295	7,080	24.00	CHINA	7	263	37.57	AUSTRALIA	8	413	51.62
UNITED KINGDOM	257	14,345	55.82	CANADA	6	92	15.33	FRANCE	3	19	6.33
CANADA	202	5,382	26.64	KOREA	5	167	33.40	PORTUGAL	3	159	53.00

**FIGURE 3:**
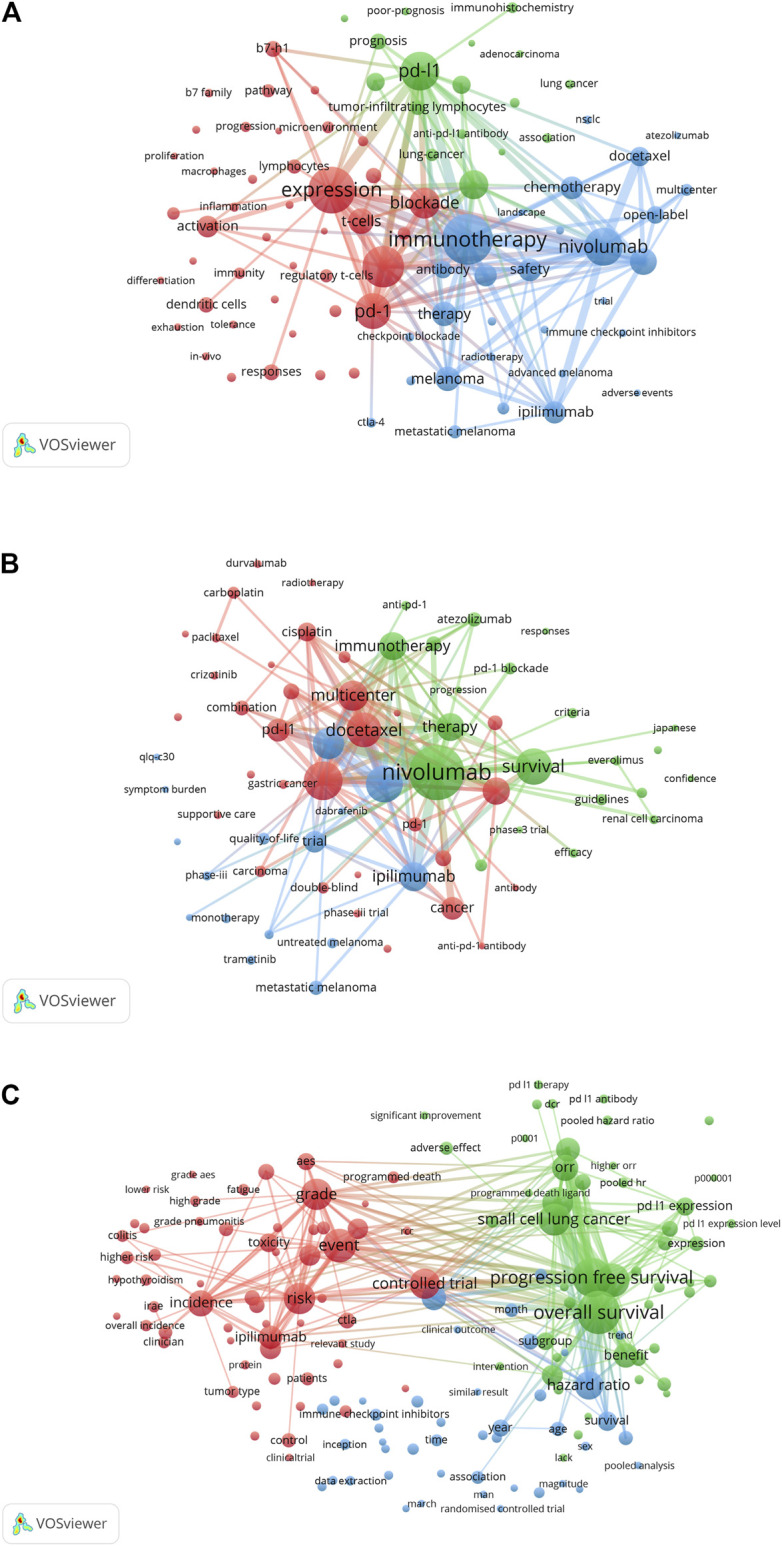
VOSviewer network visualization map of keyword and term co-occurrence analyses. The cluster analysis of keyword co-occurrence in the articles on the PD1/PDL1 molecule **(A)** and RCT of anti-PD1/PDL1 **(B)**. **(C)** The cluster analysis of term co-occurrence in meta-analysis of anti-PD1/PDL1.

The co-occurrence analysis was then conducted on the keywords (author keywords and Keywords Plus) and terms (title and abstract fields). The use of keywords and terms with a high frequency of occurrence could indicate their importance in the research fields. As for the 11,971 documents covering the PD1/PDL1 molecule, when the minimum number of occurrences of a keyword was set to 200, 97 keywords met the threshold and formed three clusters (Figure 3A). Cluster 1 (shown in red) was mainly related to expression and mechanism such as PD1, B7-H1 (PDL1), activation, response, pathway, and lymphocytes. Cluster 2 (blue) consisted of the RCT of immunotherapy including nivolumab, ipilimumab, open-label, multi-center, safety, chemotherapy, and docetaxel, which was consistent with the keyword co-occurrence analysis of RCT (Figure 3B). Cluster 3 (green) was composed largely of cancers, survival, and biomarkers covering lung-cancer, prognosis, microsatellite instability, and mutations.

Our study indicated that the studies of PD1/PDL1 molecule were relatively comprehensive. The biomarkers used for predicting the efficacy of anti-PD1/PDL1 are essential components in the Cluster 3 (green), and also necessary for the selection of patients most likely to respond to ICB from overall individuals; this could decrease the cost of treatment and avoid irAEs (Yi et al., 2018). Biomarkers including mismatch repair deficiency (dMMR), MSI-H, tumor mutational burden (TMB), quantity of tumor infiltrating lymphocyte (TIL), and so forth were identified as valuable predictors (Teng et al., 2018; Yan et al., 2018; Zhao et al., 2019; Ballot et al., 2020). However, a recent study suggested that high TMB could not predict the response to ICB across all cancer types, which indicated that further tumor type-specific studies for biomarkers should be conducted (McGrail et al., 2021). Besides, researchers have demonstrated that prior antibiotic therapy resulted in a poorer treatment response and OS in unselected patients treated with ICB (Pinato et al., 2019). The article captured the attention and interest of scientists and contributed to several further meta-analyses (Lurienne et al., 2020; Wilson et al., 2020; Yang et al., 2020), which indicated the ability of meta-analysis to reflect and explore key issues in the field. Beyond that, researchers have explored the mechanisms of resistance of matters such as insufficient tumor immunogenicity, irreversible T cell exhaustion, disfunction of MHC, immunosuppressive microenvironment, and some oncogenic signaling pathways to improve anti-PD1/PDL1 efficacy (Chocarro de Erauso et al., 2020; Lei et al., 2020; Sun et al., 2020).

We further analyzed keyword co-occurrence in RCT and term co-occurrence in meta-analysis of anti-PD1/PDL1. The 69 keywords occurring at least five times and 155 terms occurring 10 times were included, respectively. As for the keyword co-occurrence analysis in RCT, the three clusters were related to the therapy strategies of different malignancies. The red cluster was the largest and it displayed the treatments and medications used to combine, or be compared, with anti-PD1/PDL1 in lung cancer, including chemotherapy, radiotherapy, docetaxel, cisplatin, carboplatin, paclitaxel, gefitinib, crizotinib and so forth. The other two clusters referred to renal cell carcinoma (sorafenib and everlimus), melanoma (trametinib) and the quality of life of patients, respectively (Figure 3B). The three clusters of term co-occurrence analysis in meta-analysis were composed largely of irAEs, survival, and benefits with anti-PD1/PDL1, and technical terms pertaining to meta-analysis (Figure 3C).

The top 10 contributors in terms of journal among the three datasets are listed in Table 3. Consistent with our expectations, the RCT of anti-PD1/PDL1 were mostly published in the top medical journals such as New England Journal of Medicine, Lancet Oncology, Lancet, Annals of Oncology, Journal of Clinical Oncology, Journal of Thoracic Oncology, *etc*. Our analysis found that excellent meta-analyses of anti-PD1/PDL1 were likely to be accepted in some influential journals such as JAMA Oncology which was the seventh top contributor (11 of 335 documents). Besides, the other journals had more than nine published documents and were significant for researchers in the field as first choices for submitting their meta-analysis; the results pertaining to the PD1/PDL1 molecule were more persuasive because of the large number of publications: 2,678 articles have been published in the top 10 journals which account for 22.37% of the total publications. For authors, articles related to PD1/PDL1 molecule are more likely to be accepted by these journals, as they have previously shown significant interest.

**TABLE 3:** Top 10 contributors in terms of journal among the three datasets.

PD1/PDL1 molecule			RCT of anti-PD1/PDL1			Meta-analysis of anti-PD1/PDL1		
Source	IF	Records	Source	IF	Records	Source	IF	Records
ONCOIMMUNOLOGY	5.869	445	NEW ENGLAND JOURNAL OF MEDICINE	74.699	28	IMMUNOTHERAPY	2.964	19
CLINICAL CANCER RESEARCH	10.107	396	LANCET ONCOLOGY	33.752	26	MEDICINE	1.552	17
ONCOTARGET	-	304	LANCET	60.392	13	INTERNATIONAL IMMUNOPHARMACOLOGY	3.943	13
JOURNAL FOR IMMUNOTHERAPY OF CANCER	9.913	296	ANNALS OF ONCOLOGY	18.274	11	ONCOTARGET	-	13
CANCER IMMUNOLOGY RESEARCH	8.728	284	JOURNAL OF CLINICAL ONCOLOGY	32.956	10	ONCOTARGETS AND THERAPY	3.337	12
CANCER IMMUNOLOGY IMMUNOTHERAPY	5.442	233	JOURNAL OF THORACIC ONCOLOGY	13.357	10	FRONTIERS IN PHARMACOLOGY	4.225	11
CANCER RESEARCH	9.727	209	EUROPEAN JOURNAL OF CANCER	7.275	9	JAMA ONCOLOGY	24.799	11
PLOS ONE	2.74	176	FUTURE ONCOLOGY	2.66	8	CRITICAL REVIEWS IN ONCOLOGY HEMATOLOGY	5.833	10
SCIENTIFIC REPORTS	3.998	171	JAMA ONCOLOGY	24.799	8	ONCOIMMUNOLOGY	5.869	10
FRONTIERS IN IMMUNOLOGY	5.085	164	NATURE MEDICINE	36.13	6	CANCER MEDICINE	3.491	9

The top 15 most highly cited articles of RCT and meta-analysis of anti-PD1/PDL1 sorted by LCS are listed in Tables 4, 5, respectively. Our analysis found that the top 15 highly cited articles sorted by GCS were inconsistent with the ranking as ordered by LCS (Supplementary Tables S2, S3). As for RCT, the top 15 GCS articles were all cited more than 1,000 times, compared with only the top four LCS articles. This indicated that there was a difference between the articles that were more valuable to other fields and those generating the most concern in the current field. All of the top 15 articles in these two datasets were published before 2019 (perhaps because the most recently published articles have yet to be fully cited). Twelve in 15 top LCS articles were published in Lancet, Lancet Oncology, and New England Journal of Medicine (the most frequent source journal in ten of 15 top GCS articles). These were also the top three contributors with regard to journal in the RCT of anti-PD1/PDL1 (Table 3). Besides, the top 15 most highly cited documents of PD1/PDL1 molecule sorted by LCS and GCS were listed in Supplementary Tables S4, S5.

**TABLE 4:** Top 15 RCT of anti-PD1/PDL1 sorted by LCS.

Title	Source	Year	LCS	GCS
Pembrolizumab versus docetaxel for previously treated, PD-L1-positive, advanced non-small-cell lung cancer (KEYNOTE-010): a randomised controlled trial	HERBST RS, LANCET	2016	37	2,399
Atezolizumab versus docetaxel in patients with previously treated non-small-cell lung cancer (OAK): a phase 3, open-label, multicentre randomised controlled trial	RITTMEYER A, LANCET	2017	27	1,426
Atezolizumab versus docetaxel for patients with previously treated non-small-cell lung cancer (POPLAR): a multicentre, open-label, phase 2 randomised controlled trial	FEHRENBACHER L, LANCET	2016	18	1,151
Nivolumab versus chemotherapy in patients with advanced melanoma who progressed after anti-CTLA-4 treatment (CheckMate 037): a randomised, controlled, open-label, phase 3 trial	WEBER JS, LANCET ONCOL	2015	13	1,331
Combined nivolumab and ipilimumab versus ipilimumab alone in patients with advanced melanoma: 2-years overall survival outcomes in a multicentre, randomised, controlled, phase 2 trial	HODI FS, LANCET ONCOL	2016	11	392
Nivolumab in patients with advanced gastric or gastro-oesophageal junction cancer refractory to, or intolerant of, at least two previous chemotherapy regimens (ONO-4538–12, ATTRACTION-2): a randomised, double-blind, placebo-controlled, phase 3 trial	KANG YK, LANCET	2017	11	431
Pembrolizumab versus investigator-choice chemotherapy for ipilimumab-refractory melanoma (KEYNOTE-002): a randomised, controlled, phase 2 trial	RIBAS A, LANCET ONCOL	2015	9	791
Carboplatin and pemetrexed with or without pembrolizumab for advanced, non-squamous non-small-cell lung cancer: a randomised, phase 2 cohort of the open-label KEYNOTE-021 study	LANGER CJ, LANCET ONCOL	2016	8	633
Health-related quality-of-life results for pembrolizumab versus chemotherapy in advanced, PD-L1-positive NSCLC (KEYNOTE-024): a multicentre, international, randomised, open-label phase 3 trial	BRAHMER JR, LANCET ONCOL	2017	7	81
Nivolumab for Metastatic Renal Cell Carcinoma: Results of a Randomized phase II Trial	MOTZER RJ, J CLIN ONCOL	2015	6	556
Quality of life in patients with advanced renal cell carcinoma given nivolumab versus everolimus in CheckMate 025: a randomised, open-label, phase 3 trial	CELLA D, LANCET ONCOL	2016	6	106
Pembrolizumab versus ipilimumab for advanced melanoma: final overall survival results of a multicentre, randomised, open-label phase 3 study (KEYNOTE-006)	SCHACHTER J, LANCET	2017	5	331
Nivolumab plus ipilimumab or nivolumab alone versus ipilimumab alone in advanced melanoma (CheckMate 067): 4-years outcomes of a multicentre, randomised, phase 3 trial	HODI FS, LANCET ONCOL	2018	5	230
Clinical activity and molecular correlates of response to atezolizumab alone or in combination with bevacizumab versus sunitinib in renal cell carcinoma	MCDERMOTT DF, NAT MED	2018	5	169
Nivolumab vs. investigator’s choice in recurrent or metastatic squamous cell carcinoma of the head and neck: 2-years long-term survival update of CheckMate 141 with analyses by tumor PD-L1 expression	FERRIS RL, ORAL ONCOL	2018	5	108

**TABLE 5:** Top 15 meta-analysis of anti-PD1/PDL1 sorted by LCS.

Title	Source	Year	LCS	GCS
Cancer immunotherapy efficacy and patients’ sex: a systematic review and meta-analysis	CONFORTI F, LANCET ONCOL	2018	19	109
Incidence of Programmed Cell Death 1 Inhibitor-Related Pneumonitis in Patients With Advanced Cancer: A Systematic Review and Meta-analysis	NISHINO M, JAMA ONCOL	2016	17	185
Risk of elevated transaminases in cancer patients treated with immune checkpoint inhibitors: a meta-analysis	ABDEL-RAHMAN O, EXPERT OPIN DRUG SAF	2015	14	30
Risk of endocrine complications in cancer patients treated with immune check point inhibitors: a meta-analysis	ABDEL-RAHMAN O, FUTURE ONCOL	2016	14	69
Clinical and Molecular Characteristics Associated With Survival Among Patients Treated With Checkpoint Inhibitors for Advanced Non-Small Cell Lung Carcinoma: A Systematic Review and Meta-analysis	LEE CK, JAMA ONCOL	2018	14	99
Risk of cutaneous toxicities in patients with solid tumors treated with immune checkpoint inhibitors: a meta-analysis	ABDEL-RAHMAN O, FUTURE ONCOL	2015	13	48
Fatal Toxic Effects Associated With Immune Checkpoint Inhibitors: A Systematic Review and Meta-analysis	WANG DY, JAMA ONCOL	2018	13	175
Safety and Tolerability of PD-1/PD-L1 Inhibitors Compared with Chemotherapy in Patients with Advanced Cancer: A Meta-Analysis	NISHIJIMA TF, ONCOLOGIST	2017	11	70
A Network Meta-analysis Comparing the Efficacy and Safety of Anti-PD-1 with Anti-PD-L1 in Non-small Cell Lung Cancer	YOU W, J CANCER	2018	10	16
Comparison of efficacy of immune checkpoint inhibitors (ICIs) between younger and older patients: A systematic review and meta-analysis	NISHIJIMA TF, CANCER TREAT REV	2016	9	88
Incidence of Endocrine Dysfunction Following the Use of Different Immune Checkpoint Inhibitor Regimens: A Systematic Review and Meta-analysis	BARROSO-SOUSA R, JAMA ONCOL	2018	9	148
Risk of pneumonitis in cancer patients treated with immune checkpoint inhibitors: a meta-analysis	ABDEL-RAHMAN O, THER ADV RESPIR DIS	2016	8	56
Checkpoint Inhibitors in Metastatic EGFR-Mutated Non-Small Cell Lung Cancer-A Meta-Analysis	LEE CK, J THORAC ONCOL	2017	8	278
Comprehensive Meta-analysis of Key Immune-Related Adverse Events from CTLA-4 and PD-1/PD-L1 Inhibitors in Cancer Patients	DE VELASCO G, CANCER IMMUNOL RES	2017	8	106
Immune checkpoint inhibitors and targeted therapies for metastatic melanoma: A network meta-analysis	PASQUALI S, CANCER TREAT REV	2017	7	23

Most of the top LCS articles in RCT focused on non-small-cell lung cancer (NSCLC, four articles) and melanoma (five articles), which might be due to the better efficacy of immunotherapy therein. Researchers also paid attention to the choice of treatment population such as refractory or PDL1-positive cancer patients. The meta-analysis reflected the controversial and unresolved issues in RCT, and the top LCS articles in meta-analysis mainly investigated the irAEs including pneumonitis, elevated transaminases, endocrine complications, and cutaneous toxicities. Furthermore, two network meta-analysis and two articles exploring the characteristics of population including gender and age were covered in the list. Women have stronger innate and adaptive immune responses than men, which results in more rapid clearance of pathogens and lower prevalence of some infections (Klein and Flanagan, 2016; vom Steeg and Klein, 2016). Considering the immune-related mechanisms of anti-PD1/PDL1, the most highly cited article of meta-analysis sorted by LCS (Table 5, 19 times in 335 documents) included 20 randomized controlled trials and indicated a greater efficacy of ICB compared with the standard of care for men than women (Conforti et al., 2018). This attracted extensive attention and contributed to several related research projects. The meta-analysis published in JAMA Oncology found no significant sex-associated differences in the efficacy of ICB for overall and various sub-group analyses in 2019 (Wallis et al., 2019). The further analysis of Conforti et al. (2019) suggested that anti-PD1 alone had a greater advantage in men while anti-PD1/PDL1 plus chemotherapy was more effective among women. Because meta-analysis did not solve the concern of researchers, a study focused on sex-associated molecular differences for cancer immunotherapy responsiveness and suggested it was associated with cancer types, such as male-bias in melanoma and female-bias in LUSC (Ye et al., 2020). Recently, this research team explored the association between sex and irAEs and the results indicated that minimal sex-associated differences in irAEs which might be unnecessary to consider it (Jing et al., 2021). Whether the biological difference of sex could be the variable affecting the treatment benefits of anti-PD1/PDL1 is currently unclear and warrants further research.

**FIGURE 4:**
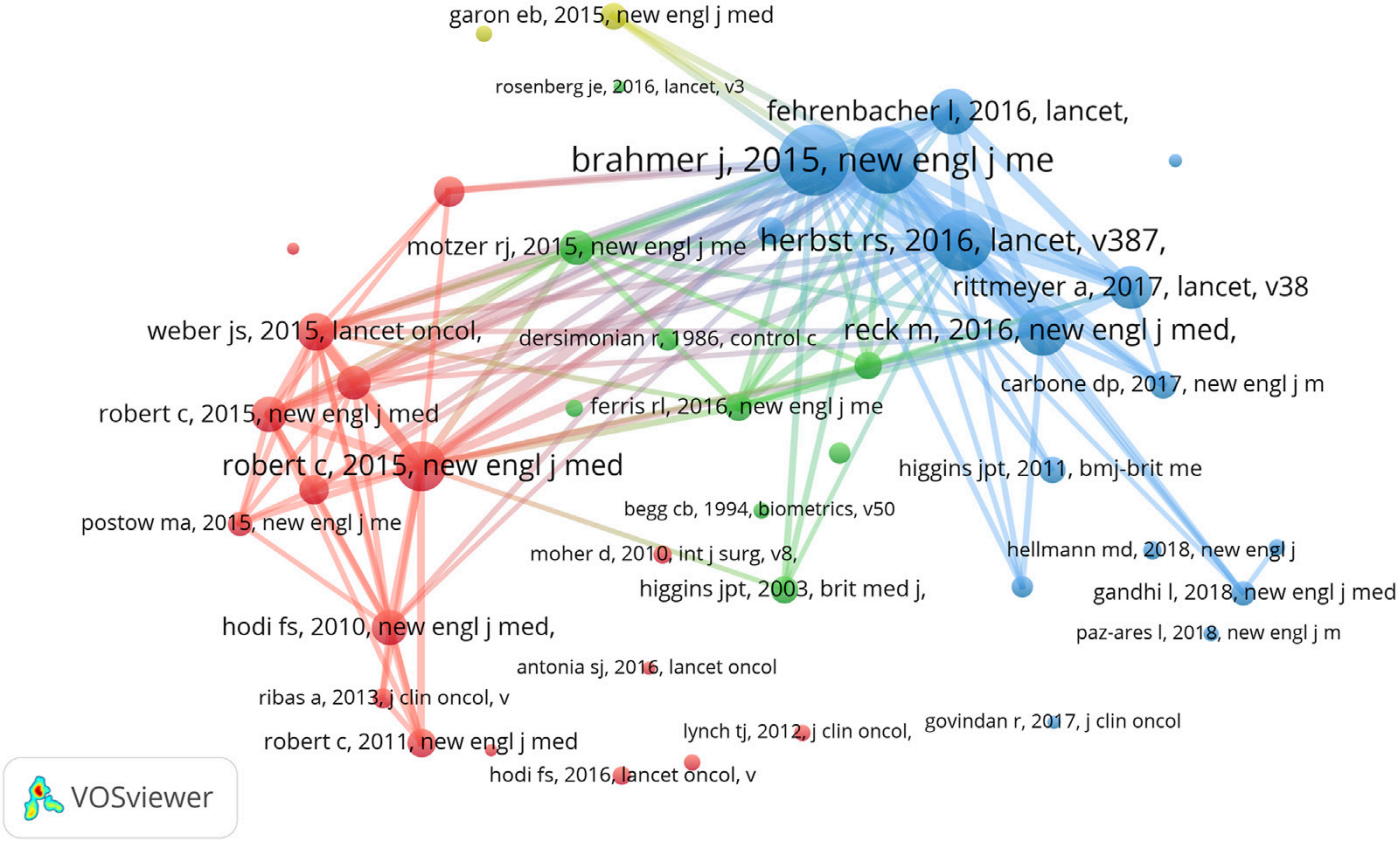
VOSviewer network visualization map of co-citation analysis in meta-analysis of anti-PD1/PDL1.

We also found that Omar Abdel-Rahman from Ain Shams University (as first author) contributed 12 meta-analyses of anti-PD1/PDL1 in which three articles were included in the top 15 LCS articles. Considering the correlation between RCT and meta-analysis, we conducted co-citation analysis in the meta-analysis dataset (Figure 4). When the minimum number of citations of a cited reference was set to 30, 44 of 6,033 references met the threshold. Consistent with our speculation, the highly co-cited references of meta-analyses could be found among the list of the top 15 GCS articles in RCT.

### The Historical Evolution of PD1/PDL1 and anti-PD1/PDL1

**FIGURE 5:**
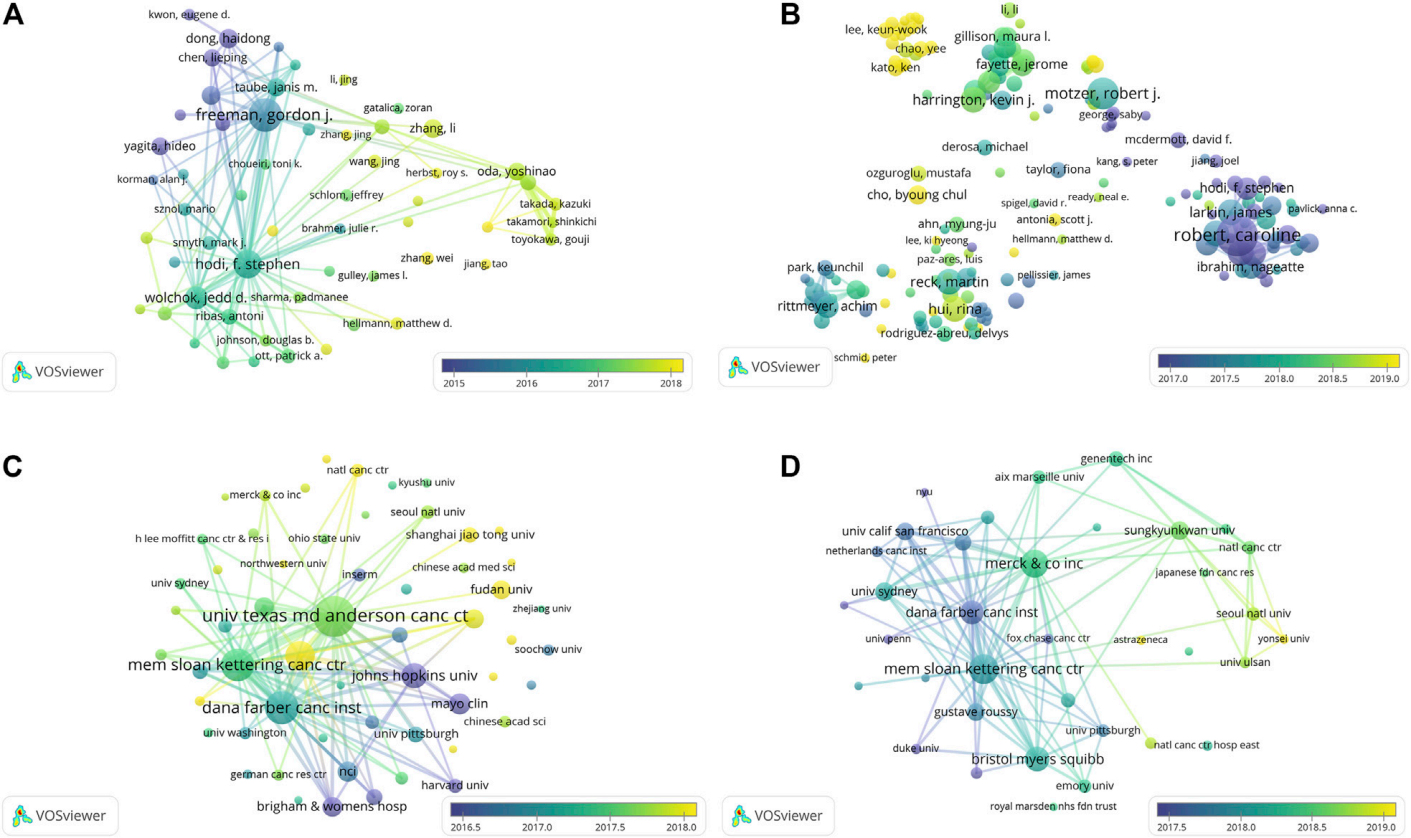
The overlay visualization map of author and institution coupling analyses. Author coupling analysis **(A)** and institution coupling analysis **(C)** for work on the PD1/PDL1 molecule. Author coupling analysis **(B)** and institution coupling analysis **(D)** in RCT of anti-PD1/PDL1.

A bibliographic coupling overlay was applied for work on the PD1/PDL1 molecule and RCT of anti-PD1/PDL1; the color of each node represents the average year of publication (Figure 5). The authors or organizations with a coupling relationship cited the same literature, indicating the relevance of their research. In the articles on the PD1/PDL1 molecule, we found the active authors changing over time. Gordon J Freeman shown in purple and F Stephen Hodi in aquamarine led the field in its early stage. Nowadays, more scientists from a wider range of countries have participated in exploration of the PD1/PDL1 molecule: these newer researchers shown in yellow represent a significant portion authors, which illustrated the good prospects for research therein (Figure 5A). As shown in Figure 5C, Johns Hopkins University, Mayo Clinic, and Harvard University have been working on the PD1/PDL1 molecule since the initiation of research into this subject. University of Texas MD Anderson Cancer Center and Dana-Farber Cancer Institute were more productive in about mid-2017. Recently, Sun Yat-Sen University was the major contributor, while newly active organizations were finite. This may indicate that the organizations require to pay more attention to the field. The network visualization map depicting this bibliographic coupling overlay in RCT of anti-PD1/PDL1 presented a similar trend.

**FIGURE 6:**
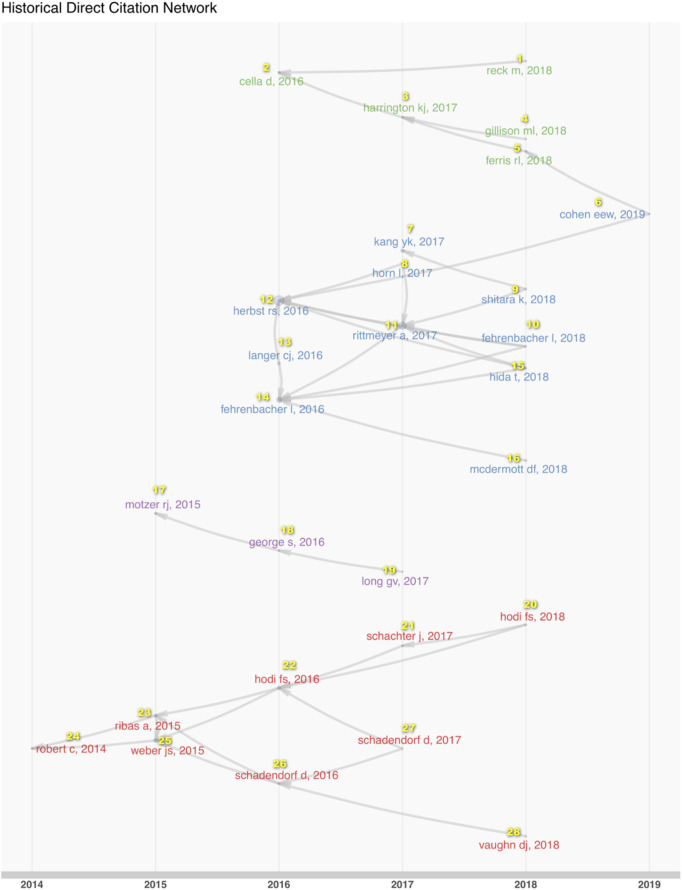
The historical direct citation network in RCT of anti-PD1/PDL1. The links among documents represented the citation relationships in the dataset.

To explore the systemic changes in the content of such research, we analyzed the historical direct citation network in RCT of anti-PD1/PDL1 (Figure 6; Supplementary Table S6). The researchers conducted RCT of anti-PD1/PDL1 in the various cancer types at different times. Melanoma was the focus upon initiation of the work in 2014, followed by renal cell carcinoma, and lung cancer sub-types. Gastric or gastroesophageal junction cancer and head and neck squamous carcinoma were paid more attention in 2017. The ICB RCT of urothelial cancer was conducted in 2019. In the middle of the process, scientists became concerned with the quality of life and the clinical benefit beyond progression issues in 2017. The studies of melanoma reflected the transformations of therapy strategies in the experimental group and the control group of RCT to a more complete extent. Anti-PD1/PDL1 was used to treat advanced and ipilimumab-refractory melanoma in earlier research. With increasing confidence in anti-PD1/PDL1 treatment, researchers contrasted combined nivolumab and ipilimumab with ipilimumab. Anti-PD1/PDL1 alone has been chosen to compare with ipilimumab in 2017.

The references with citation bursts in three datasets could also contribute to predicting the future focus for such work. The threshold was set to the top 30 per slice and references with a minimum burst duration of 2 years in RCT and meta-analysis of anti-PD1/PDL1 were displayed in Supplementary Materials S2, S3, respectively. When the minimum duration was set to 3 years, the top 100 references with the strongest citation bursts were selected from among the documents about the PD1/PDL1 molecule (Supplementary Material S4). The top six references with citation bursts in the RCT dataset appeared in 2015 and 2016 and consisted of two guidelines and four melanoma research, which indicated melanoma occupied a position of vital importance in the field. As shown in Supplementary Material S3, the top 14 references on meta-analysis could be divided into three stages by the onset of the corresponding citation bursts. The safety and activity of anti-PD1/PDL1 were of greatest concern in the first stage. Melanoma research contributed the strongest citation bursts in the second stage in 2017. Besides, two of 14 references in the third stage have the longest duration of citation burst (highly cited in 2018–2020). This suggested that ICB plus chemotherapy in lung cancer might be paid continuous attention in meta-analysis of anti-PD1/PDL1.

The highly cited references and documents contained much valuable information: we found most references were highly cited one or 2 years after publication and the citation bursts lasted no more than 5 years. The citation bursts coincided with the publication and lasted for more years in only a few cases, which might represent the major milestones in the development of PD1/PDL1-related research. Ten of the top 100 references with the strongest citation bursts in documents about the PD1/PDL1 molecule followed the rules and mainly focus on the molecular mechanism of action of PD1/PDL1 on T cells in the tumor microenvironment (Supplementary Material S4) (Carter et al., 2002; Dong et al., 2002; Blank et al., 2004). These reports involved the first identification of PDL2 (the second ligand for PD1 with the overlapping functions of PDL1) and B7-Dc (a new dendritic cell molecule with potent costimulatory properties for T cells) in 2001 (Latchman et al., 2001; Tseng et al., 2001). The last one appeared in 2005 and revealed that the expression of PDL1 in mouse cancers conferred resistance to immunotherapy by anti-CD137 activity, and blockading of PD1/PDL1 could reverse the resistance and enhance the therapeutic efficacy thereof (Hirano et al., 2005).

### The Developmental Trends of PD1/PDL1 and anti-PD1/PDL1

**FIGURE 7:**
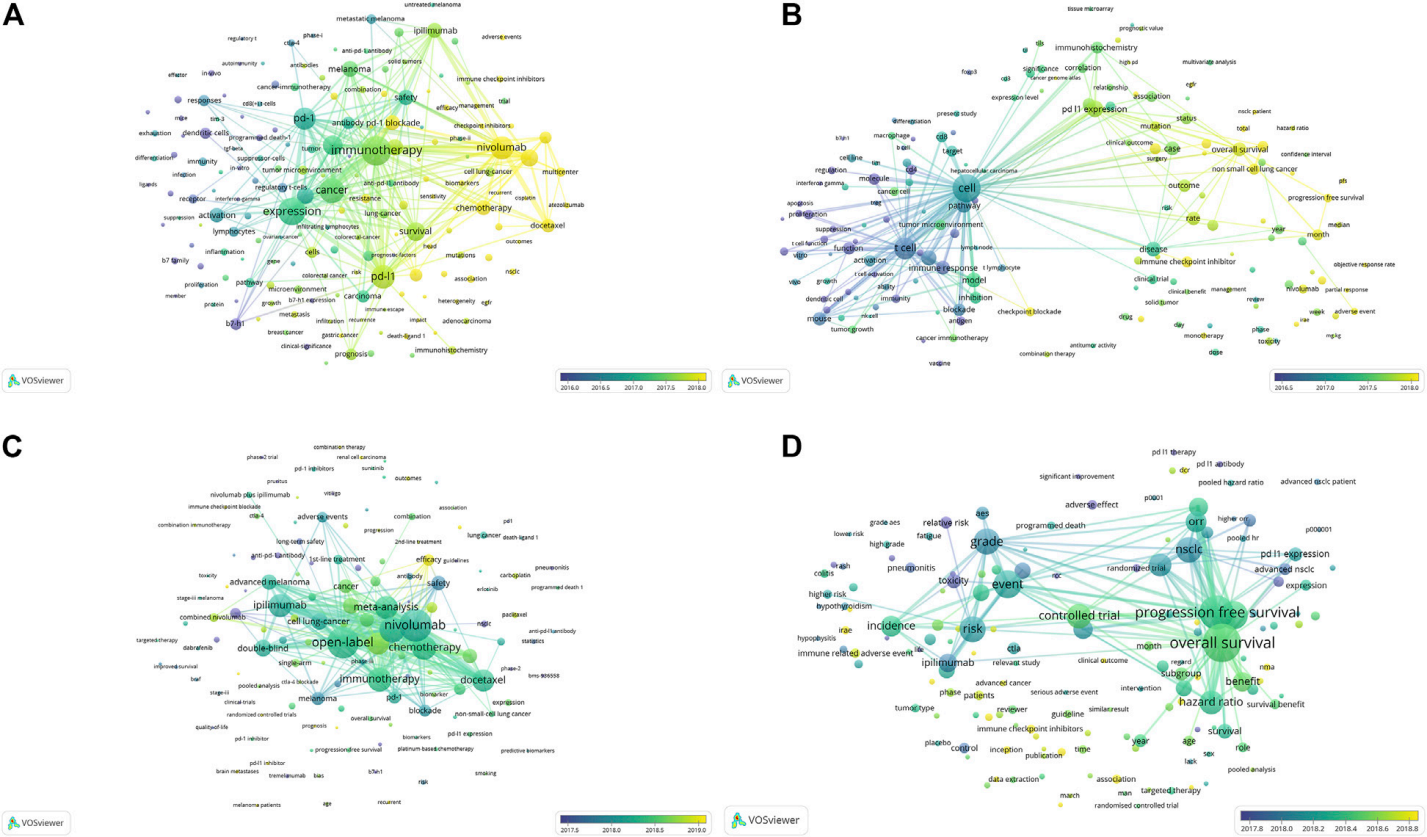
The overlay visualization map of keyword and term co-occurrence analyses.Keyword co-occurrence overlay **(A)** and term co-occurrence overlay **(B)** in the articles on the PD1/PDL1 molecule. Keyword co-occurrence overlay **(C)** and term co-occurrence overlay **(D)** in meta-analysis of anti-PD1/PDL1.

Keyword and term co-occurrence overlays were conducted for work on the PD1/PDL1 molecule and meta-analysis of anti-PD1/PDL1 to explore the developmental trends in the field (Figure 7). As for the PD1/PDL1 molecule, we found that the research focus changed from the molecular mechanism (in purple) to the expression of related molecules and immunotherapy (in aquamarine), following by antitumor drugs including ICB and tyrosine kinases inhibitors (TKI) from the keywords. Besides, the biomarkers of immunotherapy covering microsatellite instability (MSI), the characters of patients’ responses to anti-PD1/PDL1 treatment such as sensitivity, resistance and heterogeneity (in yellow) might warrant sustained attention in the future (Figure 7A). There was a similar trend in the analysis of the term co-occurrence overlay, and the transition from molecular mechanism to clinical characteristics (treatment, survival, and clinical benefits) was demonstrated. The close connection between clinicopathologic features (immunohistochemistry and mRNA expression) (in aquamarine) and clinical characteristics (in yellow) was shown in Figure 7B. These reflected the process from the discovery of the PD1/PDL1 molecule to the clinical application of anti-PD1/PDL1, which was in accordance with the tendency in the development of work on such molecules. In the meta-analysis of anti-PD1/PDL1, researchers might be dedicated to irAEs, the treatment strategy of combination therapy, randomized controlled trails, and work on some select sub-population such as patients with recurrent caners and brain metastases in the future (Figures 7C–D).

irAEs formed the red cluster in the term co-occurrence analysis of meta-analysis (Figure 3C) and these would also be the focus of future studies (Figure 7D). The unique side effects induced by ICB are classified as irAEs, which often differ from the classical chemotherapy-related toxicities and result from the loss of immune homeostasis and off-target effects in peripheral tissues (Martins et al., 2019). The adverse events of anti-PD1/PDL1 might be irreversible and occur in, or even after, the course of treatment, especially in those patients subject to combined therapy (Lowe et al., 2016). Interestingly, recent studies suggested that the incidence of irAEs was associated with better long-term survival and overall response rates across different malignancies treated with anti-PD1/PDL1 (Das et al., 2020; Dupont et al., 2020; Maillet et al., 2020). The researchers wondered whether elderly patients could benefit from anti-PD1/PDL1 without increasing toxicities. A pooled analysis of elderly patients (those aged 75 years and over) with advanced NSCLC with PDL1-positive have indicated that pembrolizumab not only improved OS but presented a more favorable safety profile versus chemotherapy (Nosaki et al., 2019). In the second-line therapy of NSCLC, elderly patients (those aged 75 years and over) appeared to tolerate anti-PD1/PDL1 and Grade 3 or 4 treatment-related adverse events were less frequent compared to the sub-group of patients below 65 years of age (Marur et al., 2018). These serious irAEs resulted in the interruption of treatment, while current oncological guidelines recommended permanent discontinuation of ICB for only Grade 4 irAEs (Brahmer et al., 2018). The recent cohort study indicated that 28.8% recurrence rate of the same irAE associated with discontinuation of ICB therapy and 4.4% incidence rate of a different irAE after re-challenge with the same ICB. The recurrence rate was 28.6% after anti-PD1/PDL1 monotherapy resumption (Dolladille et al., 2020). Despite resumption of ICB therapy being able to be considered for selected patients, the optimal management of irAEs still relies on early recognition to limit interruptions to treatment and avoid the risk of rare fatal outcomes.

The landscape of developmental process and trends in PD1/PDL1 molecule and anti-PD1/PDL1 could be speculated by consideration of the historical direct citation network (Figure 6) as well as keyword and term co-occurrence overlays (Figure 7): the strategy of combination therapy (in yellow) could for a focus for future research. Currently, treatments combining anti-PD1/PDL1 with other ICB, conventional chemotherapy, radiotherapy, or chimeric antigen receptor T cell (CAR-T) therapy have been reported with beneficial effects being identified therein (Chen et al., 2020; Gray et al., 2020; Sullivan et al., 2020). Besides, pembrolizumab combined with natural killer cells contributed to a better survival to advanced NSCLC patients compared with pembrolizumab alone (Lin et al., 2020). Combination therapy with TKI including EGFR-TKI gefitinib and ALK-TKI crizotinib were expected in recent years because of the limited efficacy of anti-PD1/PDL1 for EGFR mutations and ALK rearrangements patients with advanced NSCLC (Gainor et al., 2016; Lisberg et al., 2018); however, severe hepato-toxicities among patients were observed in CheckMate 370 (nivolumab plus crizotinib) and KEYNOTE-021 (pembrolizumab plus gefitinib) (Spigel et al., 2018; Yang et al., 2019). Conversely, combining anti-PD1/PDL1 with TKI therapy had a manageable safety profile and encouraging antitumor activity in other cancers, such as avelumab plus axitinib for advanced renal cell carcinoma and regorafenib plus nivolumab for advanced gastric or colorectal cancer (Motzer et al., 2019; Fukuoka et al., 2020). These indicated the feasibility of the strategy involving the combination of anti-PD1/PDL1 and TKI, and the appropriate dosages and medication orders should be explored further.

## Discussion

In the present study we analyzed the literature on the PD1/PDL1 molecule and RCT as well as meta-analysis of anti-PD1/PDL1 to map the knowledge and status of the research status, historical evolution, and developmental trends of PD1/PDL1-related research from 2000 to 2020. The publication records showed explosive growth trends (with annual percentage growth rates of 37.77, 72.12, and 62.98%, respectively) and high average citations per document in the period (Table 1). As for the country-based distribution of the literature (Table 2), the United States was the most productive country with the highest average article citations for molecule (35.3%, 60.32 citations per article) and RCT (41.6%, 440.14). China is the top contributor to meta-analysis (50.1%), while the top three countries with the highest average citations were Portugal (three articles, 53.0 citations per article), the Philippines (one, 52.0), and Australia (eight, 51.62). These suggested China should pay increased attention to the quality and attractiveness of meta-analysis. The most analyses in this study were made using a full counting system in which every author collaborating on a document would be counted once. In the case of binary counting for term co-occurrence analysis, the occurrences attribute indicated the number of documents in which a term occurred at least once (van Eck and Waltman, 2010). The fractional counting system is also a widely used method, and it counts a half for two authors with a single document (Leydesdorff and Park, 2017). The differences between two counting methods should be noticed during the bibliometric analysis.

The results presented how the research focus had changed during time from molecular mechanisms to targeting PD1/PDL1 pathway in combination with other therapies and the change process of therapy lines that anti-PD1/PDL1 were used in. In addition, biomarkers, irAEs and resistance to PD1/PDL1 blockade are currently hot research topics and would most likely keep this status also in the near future. However, this retrospective analysis might not reflect the latest trends because the significant number of documents have not been published yet due to their novelty. We noticed that anti-PD1/PDL1 had been used as neoadjuvant therapy in the documents included in dataset B. The OpACIN trial suggested the feasibility of neoadjuvant combination of ipilimumab and nivolumab for stage III melanoma patients and neoadjuvant therapy expanded more tumor-resident T cell clones than adjuvant application (Blank et al., 2018; Rozeman et al., 2021). The NCT02519322 study indicated that neoadjuvant treatment with combined ipilimumab and nivolumab yielded higher response rates (RECIST ORR 73%) but substantial toxicity (73% grade 3 trAEs) compared with neoadjuvant nivolumab in high-risk resectable melanoma patients (Amaria et al., 2018). Besides, the RCT of neoadjuvant therapy with anti-PD1/PDL1 have been designed in multiple cancers including NSCLC and gastric cancer (Bang et al., 2019).

Another emerging research direction should be paid attention in is single cell sequencing which could focus on the genome or transcriptome information at a single-cell level to reveal cell population differences and cellular evolutionary (Lei et al., 2021). Several related documents were found in our datasets due to the attention of researchers to PD1/PDL1 expression in various kinds of cells (Brummelman et al., 2018; Penter et al., 2019). A recent bibliometric study focused on single cell sequencing technologies and suggested its applications in immunology would be the next research hotspot (Wang et al., 2021). We found that in single cell sequencing had been applied in the patients with anti-PD1/PDL1 therapy to figure out the changes of cell subsets after treatments and differences between responders and non-responders, which could contribute to predict prognosis, select suitable therapy strategy and monitor the condition of diseases (Bassez et al., 2021; Gohil et al., 2021). Considering that cancer immunotherapy is characterized by targeting cells in tumor microenvironment, some cell subsets may have the similar effects on anti-PD1/PDL1 therapy of different cancers. Based on the analyses of single cell sequencing technologies, the specific gene expression signatures of cancer-associated fibroblasts (CAF) have been identified to be associated with poor responses to anti-PD1 or anti-PDL1 antibodies in breast cancer and pancreatic cancer, respectively (Dominguez et al., 2020; Kieffer et al., 2020). Besides, single cell sequencing technologies have also been used in the patients with the combined treatment and irAEs. Griffiths et al. (2020) conducted single cell RNA sequencing at serial time points during treatment of modified FOLFOX6 chemotherapy followed by a combination of chemotherapy and anti-PD1 immunotherapy for the patients with advanced gastrointestinal cancers to study the population dynamics of tumor, immune cells and immune phenotypic. Luoma et al. (2020) preformed the unique research that highlighted the mechanisms of colon inflammatory adverse events induced by checkpoint blockades (anti-PD1 and anti-CTLA4) at a single-cell level and provided opportunities for therapeutic intervention.

To our knowledge, the present study is the first comprehensive bibliometric analysis to explore the major research themes and hotspot tendencies in anti-PD1/PDL1 research from three perspectives including molecular mechanisms, RCT, and meta-analysis. However, the study has certain limitations: first, a single database (WoSCC) was used to collect publications and their bibliometric data. Second, non-English language documents were excluded from the analysis which possibly resulted in source bias. Additionally, we did not analyze the most significant authorship contributions to the three datasets because many authors with similar initials led to inaccurate counting especially for Chinese authors in the meta-analysis documents. Moreover, the cleaning process for synonyms was absent in this study, because it is hard to clean data by manual filtration due to the large volume of literatures. The effective and automatic algorithms are in the learning and designing process and expected to be used in our further studies. Anti-PD1/PDL1 are the most widely used ICB in clinical practice, beyond that, immune checkpoints such as TIM-3, LAG-3, and TIGIT are also being actively investigated (Anderson et al., 2016; Andrews et al., 2019), which awaits the attention of future bibliometric studies.

## Conclusion

The present study provided a comprehensive bibliometric analysis of the literature on anti-PD1/PDL1 from three aspects including molecular mechanisms, randomized clinical trials and meta-analysis, thus producing the academic structure reflecting the status of the research, its historical evolution, and developmental trends in related research from 2000 to 2020. The results showed that research related to anti-PD1/PDL1 is still on the raise and analyzed the main contributors in the field. It also presented how the research focus had changed during time from molecular mechanisms to targeting PD1/PDL1 pathway in combination with other immune checkpoint inhibitors, targeted therapies and conventional therapies. In addition, biomarkers, irAEs and resistance to PD1/PDL1 blockade are currently hot research topics and would most likely keep this status also in the near future. This information could contribute to readers, especially to those without deep previous knowledge of the topic, in gleaning a general overview of the landscape. The results could also be used to identify potentially relevant publications, possible collaboration partners and promising research directions.

## Data Availability

The raw data supporting the conclusion of this article will be made available by the authors, without undue reservation.
