# Supplementary material for: Trends in the Research Into Immune Checkpoint Blockade by Anti-PD1/PDL1 Antibodies in Cancer Immunotherapy: A Bibliometric Study
Source: Front Pharmacol. 2021 Aug 17;12:670900. doi: 10.3389/fphar.2021.670900 (PMC8418110; doi:10.3389/fphar.2021.670900)
Supplement: Supplementary file 1 [file DataSheet2.pdf]

Top References with the Strongest Citation Bursts

References	Year	Strength	Begin	End	2014 - 2020
Wolchok JD, 2013, NEW ENGL J MED, V369, P122, DOI	2013	2.27	**2015**	2016	
Eisenhauer EA, 2009, EUR J CANCER, V45, P228, DOI	2009	6.2734	**2015**	2017	
Wolchok JD, 2009, CLIN CANCER RES, V15, P7412, DOI	2009	2.4889	**2015**	2017	
Robert C, 2015, NEW ENGL J MED, V372, P320, DOI	2015	2.1852	**2015**	2017	
Ribas A, 2015, LANCET ONCOL, V16, P908, DOI	2015	2.2006	**2016**	2017	
Postow MA, 2015, NEW ENGL J MED, V372, P2006, DOI	2015	2.2006	**2016**	2017	
