# Supplementary material for: Trends in the Research Into Immune Checkpoint Blockade by Anti-PD1/PDL1 Antibodies in Cancer Immunotherapy: A Bibliometric Study
Source: Front Pharmacol. 2021 Aug 17;12:670900. doi: 10.3389/fphar.2021.670900 (PMC8418110; doi:10.3389/fphar.2021.670900)
Supplement: Supplementary file 3 [file DataSheet4.pdf]

Top 100 References with the Strongest Citation Bursts

References	Year	Strength	Begin	End	2000 - 2020
Nishimura H, 1999, IMMUNITY, V11, P141, DOI	1999	9.9607	**2001**	2004	
Latchman Y, 2001, NAT IMMUNOL, V2, P261, DOI	2001	21.9322	**2001**	2006	
Tseng SY, 2001, J EXP MED, V193, P839, DOI	2001	14.8252	**2001**	2006	
Wang SD, 2000, BLOOD, V96, P2808	2000	3.3585	**2001**	2003	
Nishimura H, 2001, SCIENCE, V291, P319, DOI	2001	17.4083	**2001**	2006	
Freeman GJ, 2000, J EXP MED, V192, P1027, DOI	2000	25.5619	**2001**	2005	
Tamura H, 2001, BLOOD, V97, P1809, DOI	2001	9.2961	**2001**	2004	
Dong HD, 1999, NAT MED, V5, P1365	1999	19.276	**2001**	2004	
Yoshinaga SK, 1999, NATURE, V402, P827, DOI	1999	4.0305	**2001**	2003	
Carter LL, 2002, EUR J IMMUNOL, V32, P634, DOI	2002	9.5336	**2002**	2007	
Dong HD, 2002, NAT MED, V8, P793, DOI	2002	48.5068	**2002**	2007	
Nishimura H, 2001, TRENDS IMMUNOL, V22, P265, DOI	2001	13.0781	**2002**	2006	
Sharpe AH, 2002, NAT REV IMMUNOL, V2, P116, DOI	2002	11.1141	**2002**	2006	
Carreno BM, 2002, ANNU REV IMMUNOL, V20, P29, DOI	2002	9.8052	**2002**	2006	
Ishida M, 2002, IMMUNOL LETT, V84, P57, DOI	2002	5.9718	**2002**	2005	
Chambers CA, 2001, ANNU REV IMMUNOL, V19, P565, DOI	2001	5.2269	**2002**	2006	
Iwai Y, 2002, P NATL ACAD SCI USA, V99, P12293, DOI	2002	31.453	**2003**	2007	
Wang SD, 2003, J EXP MED, V197, P1083, DOI	2003	10.0295	**2003**	2005	
Mazanet MM, 2002, J IMMUNOL, V169, P3581, DOI	2002	10.5368	**2003**	2006	
Curiel TJ, 2003, NAT MED, V9, P562, DOI	2003	30.2665	**2003**	2008	
Chapoval AI, 2001, NAT IMMUNOL, V2, P269, DOI	2001	6.0152	**2003**	2005	
Brown JA, 2003, J IMMUNOL, V170, P1257, DOI	2003	27.7366	**2003**	2008	
Strome SE, 2003, CANCER RES, V63, P6501	2003	28.0632	**2004**	2008	
Phan GQ, 2003, P NATL ACAD SCI USA, V100, P8372, DOI	2003	5.1799	**2004**	2007	
Prasad DVR, 2003, IMMUNITY, V18, P863, DOI	2003	6.6568	**2004**	2006	
Yamazaki T, 2002, J IMMUNOL, V169, P5538, DOI	2002	13.6095	**2004**	2007	
Blank C, 2004, CANCER RES, V64, P1140, DOI	2004	19.3315	**2004**	2009	
Sica GL, 2003, IMMUNITY, V18, P849, DOI	2003	8.6557	**2004**	2006	
Ansari MJI, 2003, J EXP MED, V198, P63, DOI	2003	6.3631	**2004**	2008	
Dong HD, 2004, IMMUNITY, V20, P327, DOI	2004	11.2147	**2004**	2009	
Wintterle S, 2003, CANCER RES, V63, P7462	2003	22.3091	**2004**	2008	
Dong HD, 2003, J CLIN INVEST, V111, P363, DOI	2003	7.9893	**2004**	2006	
Iwai YH, 2003, J EXP MED, V198, P39, DOI	2003	9.0686	**2004**	2007	
Liang SC, 2003, EUR J IMMUNOL, V33, P2706, DOI	2003	6.4758	**2004**	2007	
Youngnak P, 2003, BIOCHEM BIOPH RES CO, V307, P672, DOI	2003	3.8844	**2004**	2007	
Zang XX, 2003, P NATL ACAD SCI USA, V100, P10388, DOI	2003	4.6588	**2004**	2006	
Subudhi SK, 2004, J CLIN INVEST, V113, P694, DOI	2004	3.993	**2004**	2006	
Dong HD, 2003, J MOL MED, V81, P281, DOI	2003	21.0314	**2004**	2008	
Hirano F, 2005, CANCER RES, V65, P1089	2005	37.4863	**2005**	2010	
Chen LP, 2004, NAT REV IMMUNOL, V4, P336, DOI	2004	25.2848	**2005**	2009	
Konishi J, 2004, CLIN CANCER RES, V10, P5094, DOI	2004	20.5274	**2006**	2009	
Ohigashi Y, 2005, CLIN CANCER RES, V11, P2947, DOI	2005	21.8134	**2006**	2009	
Curiel TJ, 2004, NAT MED, V10, P942, DOI	2004	8.9696	**2006**	2009	
Thompson RH, 2004, P NATL ACAD SCI USA, V101, P17174, DOI	2004	25.0299	**2006**	2009	
Greenwald RJ, 2005, ANNU REV IMMUNOL, V23, P515, DOI	2005	36.8263	**2006**	2010	
Ghebeh H, 2006, NEOPLASIA, V8, P190, DOI	2006	7.5078	**2007**	2011	
Barber DL, 2006, NATURE, V439, P682, DOI	2006	54.7131	**2007**	2011	
Thompson RH, 2006, CANCER RES, V66, P3381, DOI	2006	28.2181	**2007**	2011	
Latchman YE, 2004, P NATL ACAD SCI USA, V101, P10691, DOI	2004	11.7179	**2007**	2009	

Keir ME, 2006, J EXP MED, V203, P883, DOI	2006	17.5374	**2007**	2011
Dorfman DM, 2006, AM J SURG PATHOL, V30, P802, DOI	2006	18.165	**2007**	2011
Probst HC, 2005, NAT IMMUNOL, V6, P280, DOI	2005	6.5064	**2007**	2009
Petrovas C, 2006, J EXP MED, V203, P2281, DOI	2006	16.4553	**2007**	2010
Trautmann L, 2006, NAT MED, V12, P1198, DOI	2006	27.5892	**2007**	2011
Blank C, 2005, CANCER IMMUNOL IMMUN, V54, P307, DOI	2005	12.3698	**2007**	2009
Blank C, 2006, INT J CANCER, V119, P317, DOI	2006	20.6762	**2007**	2011
Parry RV, 2005, MOL CELL BIOL, V25, P9543, DOI	2005	6.322	**2007**	2010
Zou WP, 2005, NAT REV CANCER, V5, P263, DOI	2005	7.1575	**2007**	2009
Day CL, 2006, NATURE, V443, P350, DOI	2006	37.0328	**2007**	2011
Thompson RH, 2007, CLIN CANCER RES, V13, P1757, DOI	2007	5.8523	**2008**	2010
Liu JZ, 2007, BLOOD, V110, P296, DOI	2007	9.0138	**2008**	2011
Urbani S, 2006, J VIROL, V80, P11398, DOI	2006	17.3993	**2008**	2011
Blank C, 2007, CANCER IMMUNOL IMMUN, V56, P739, DOI	2007	10.6243	**2008**	2012
Butte MJ, 2007, IMMUNITY, V27, P111, DOI	2007	20.6462	**2008**	2012
Azuma T, 2008, BLOOD, V111, P3635, DOI	2008	9.8274	**2009**	2011
Zou WP, 2008, NAT REV IMMUNOL, V8, P467, DOI	2008	25.292	**2009**	2013
Sharpe AH, 2007, NAT IMMUNOL, V8, P239, DOI	2007	24.2118	**2009**	2012
Roncador G, 2007, HAEMATOLOGICA, V92, P1059, DOI	2007	11.7954	**2009**	2011
Keir ME, 2008, ANNU REV IMMUNOL, V26, P677, DOI	2008	75.7052	**2009**	2013
Hamanishi J, 2007, P NATL ACAD SCI USA, V104, P3360, DOI	2007	15.277	**2009**	2012
Nomi T, 2007, CLIN CANCER RES, V13, P2151, DOI	2007	14.6394	**2009**	2012
de Leval L, 2007, BLOOD, V109, P4952, DOI	2007	12.0901	**2009**	2012
Blackburn SD, 2009, NAT IMMUNOL, V10, P29, DOI	2009	28.3377	**2010**	2014
Berger R, 2008, CLIN CANCER RES, V14, P3044, DOI	2008	19.0764	**2010**	2013
Xerri L, 2008, HUM PATHOL, V39, P1050, DOI	2008	7.7956	**2010**	2012
Ahmadzadeh M, 2009, BLOOD, V114, P1537, DOI	2009	31.3003	**2010**	2014
Gao Q, 2009, CLIN CANCER RES, V15, P971, DOI	2009	16.5077	**2010**	2014
Francisco LM, 2009, J EXP MED, V206, P3015, DOI	2009	20.0716	**2011**	2014
Brahmer JR, 2010, J CLIN ONCOL, V28, P3167, DOI	2010	60.5392	**2011**	2015
Velu V, 2009, NATURE, V458, P206, DOI	2009	12.6671	**2011**	2013
Fourcade J, 2010, J EXP MED, V207, P2175, DOI	2010	26.2237	**2012**	2015
Wherry EJ, 2011, NAT IMMUNOL, V12, P492, DOI	2011	38.2504	**2012**	2015
Jin HT, 2010, P NATL ACAD SCI USA, V107, P14733, DOI	2010	14.1325	**2012**	2014
Gabrilovich DI, 2009, NAT REV IMMUNOL, V9, P162, DOI	2009	7.9825	**2012**	2014
Curran MA, 2010, P NATL ACAD SCI USA, V107, P4275, DOI	2010	31.3738	**2012**	2015
Hodi FS, 2010, NEW ENGL J MED, V363, P711, DOI	2010	91.35	**2012**	2015
Hino R, 2010, CANCER-AM CANCER SOC, V116, P1757, DOI	2010	17.6544	**2012**	2015
Robert C, 2011, NEW ENGL J MED, V364, P2517, DOI	2011	52.7335	**2012**	2016
Sakuishi K, 2010, J EXP MED, V207, P2187, DOI	2010	32.5191	**2012**	2015
Topalian SL, 2012, CURR OPIN IMMUNOL, V24, P207, DOI	2012	33.0144	**2013**	2015
Topalian SL, 2012, NEW ENGL J MED, V366, P2443, DOI	2012	229.3384	**2013**	2017
Pardoll DM, 2012, NAT REV CANCER, V12, P252, DOI	2012	135.0377	**2013**	2017
Taube JM, 2012, SCI TRANSL MED, V4, P0, DOI	2012	70.3759	**2013**	2017
Schreiber RD, 2011, SCIENCE, V331, P1565, DOI	2011	22.9654	**2013**	2015
Brahmer JR, 2012, NEW ENGL J MED, V366, P2455, DOI	2012	147.6627	**2013**	2017
Woo SR, 2012, CANCER RES, V72, P917, DOI	2012	17.0651	**2013**	2015
Wolchok JD, 2013, NEW ENGL J MED, V369, P122, DOI	2013	42.4843	**2014**	2016
Hamid O, 2013, NEW ENGL J MED, V369, P134, DOI	2013	66.2803	**2014**	2016
Powles T, 2014, NATURE, V515, P558, DOI	2014	32.1831	**2015**	2018
Fehrenbacher L, 2016, LANCET, V387, P1837, DOI	2016	28.2421	**2017**	2020
