# Supplementary material for: Trends in the Research Into Immune Checkpoint Blockade by Anti-PD1/PDL1 Antibodies in Cancer Immunotherapy: A Bibliometric Study
Source: Front Pharmacol. 2021 Aug 17;12:670900. doi: 10.3389/fphar.2021.670900 (PMC8418110; doi:10.3389/fphar.2021.670900)
Supplement: Supplementary file 4 [file DataSheet3.pdf]

Top References with the Strongest Citation Bursts

References	Year	Strength	Begin	End	2014 - 2020
Hamid O, 2013, NEW ENGL J MED, V369, P134, DOI	2013	2.7402	**2014**	2015	
Wolchok JD, 2009, CLIN CANCER RES, V15, P7412, DOI	2009	3.1967	**2014**	2017	
Hodi FS, 2010, NEW ENGL J MED, V363, P711, DOI	2010	2.7016	**2014**	2017	
Topalian SL, 2014, J CLIN ONCOL, V32, P1020, DOI	2014	2.1893	**2014**	2015	
Topalian SL, 2012, NEW ENGL J MED, V366, P2443, DOI	2012	4.0866	**2014**	2015	
Chapman PB, 2011, NEW ENGL J MED, V364, P2507, DOI	2011	2.7402	**2014**	2015	
Eisenhauer EA, 2009, EUR J CANCER, V45, P228, DOI	2009	7.6084	**2014**	2017	
Robert C, 2011, NEW ENGL J MED, V364, P2517, DOI	2011	2.3653	**2015**	2017	
Robert C, 2015, NEW ENGL J MED, V372, P320, DOI	2015	3.6073	**2015**	2017	
Brahmer JR, 2010, J CLIN ONCOL, V28, P3167, DOI	2010	2.9242	**2015**	2016	
Robert C, 2015, NEW ENGL J MED, V372, P2521, DOI	2015	2.4558	**2016**	2017	
Ribas A, 2015, LANCET ONCOL, V16, P908, DOI	2015	2.4856	**2016**	2017	
Postow MA, 2015, NEW ENGL J MED, V372, P2006, DOI	2015	2.4856	**2016**	2017	
Rosenberg JE, 2016, LANCET, V387, P1909, DOI	2016	2.5556	**2017**	2018	
