# Supplementary material for: Trends in the Research Into Immune Checkpoint Blockade by Anti-PD1/PDL1 Antibodies in Cancer Immunotherapy: A Bibliometric Study
Source: Front Pharmacol. 2021 Aug 17;12:670900. doi: 10.3389/fphar.2021.670900 (PMC8418110; doi:10.3389/fphar.2021.670900)
Supplement: Supplementary file 6 [file DataSheet1.pdf]

1. **The search strategy of the PD1/PDL1 molecule (dataset A):**

TS=(((Neoplasms OR Neoplasia OR Neoplasias OR Neoplasm OR Tumors OR Tumor OR Cancer OR Cancers OR carcinoma* OR Malignancy OR Malignancies OR Malignant OR Melanoma* OR Myeloma* OR "Kahler Disease" OR Lymphoma* OR Germinoblastoma* OR *sarcoma OR *sarcomas OR leukemia* OR Leucocythaemia* OR Leucocythemia* OR Plasmocytoma* OR Plasmacytoma* OR plasmoma) AND ("Programmed Cell Death-1" OR "Programmed Cell Death Ligand-1" OR "Programmed Cell Death-Ligand 1" OR "Programmed Death-Ligand 1" OR "Programmed Death Ligand-1" OR "Programmed Death-1"OR "Programmed Cell Death protein-1" OR PD-1 OR PD-L1 OR PDL-1 OR PDL1 OR PD1 OR PDCD1LG1 OR "programmed cell death 1 ligand 1" OR B7H1 OR B7-H1 OR CD279 OR CD274)) NOT (Meta-Analyses OR Meta-Analysis OR "Meta Analysis as Topic" OR "Data Pooling*" OR "Overviews, Clinical Trial" OR "Clinical Trial Overview*" OR "Overview, Clinical Trial"))

2. **The search strategy of RCT of anti-PD1/PDL1 (dataset B):**

TS=((((checkpoint-inhibitor* OR checkpoint-block* OR checkpoint-therapy OR "immune-checkpoint inhibitor*" OR "immune-checkpoint block*" OR "immunecheckpoint inhibitor*" OR "immunecheckpoint block*" OR Nivolumab OR Opdivo OR ONO-4538 OR ONO4538 OR MDX-1106 OR MDX1106 OR BMS-936558 OR BMS936558 OR Pembrolizumab OR SCH-900475 OR SCH900475 OR Keytruda OR MK-3475 OR MK3475 OR Lambrolizumab OR Cemiplimab OR REGN2810 OR Toripalimab OR Sinitilimanb OR Camrelizumab OR SHR-1210 OR SHR1210 OR Tislelizumab OR Pidilizumab OR CT-011 OR AMP-514 OR MEDI0680 OR AMP-224 OR PDR001 OR Atezolizumab OR MSB0010718C OR RO5541267 OR MPDL-3280A OR MPDL3280A OR Tecentriq OR RG-7446 OR RG7446 OR Durvalumab OR MEDI-4736 OR MEDI4736 OR Imfinzi OR Avelumab OR MSB-0010682 OR MSB0010682 OR Bavencio OR MSB-0010718C OR MSB0010718C OR BMS-936559 OR BMS936559 OR MDX-1105 OR MDX1105 OR anti-PD-1 OR anti-PD-L1 OR anti-PDL1 OR anti-PDL-1 OR anti-PD1 OR ("Programmed Cell Death-1" OR "Programmed Cell Death Ligand-1" OR "Programmed Cell Death-Ligand 1" OR "Programmed Death-Ligand 1" OR "Programmed Death Ligand-1" OR "Programmed Death-1"OR "Programmed Cell Death protein-1" OR PD-1 OR PD-L1 OR PDL-1 OR PDL1 OR PD1 OR PDCD1LG1 OR "programmed cell death 1 ligand 1" OR B7H1 OR B7-H1 OR CD279 OR CD274)) NOT (Meta-Analyses OR Meta-Analysis OR "Meta Analysis as Topic" OR "Data Pooling*" OR "Overviews, Clinical Trial" OR "Clinical Trial Overview*" OR "Overview, Clinical Trial")) AND (random-allocation OR randomized OR randomization OR randomly OR randomisation OR randomised OR randomise OR randomize OR random)) NOT (mouse OR mice OR animal* OR rat OR rats))

3. The search strategy of meta-analysis of anti-PD1/PDL1 (dataset C):

TS=((Meta-Analyses OR Meta-Analysis OR "Meta-Analysis as Topic" OR "Data Pooling*" OR "Overviews, Clinical Trial" OR "Clinical Trial Overview*" OR "Overview, Clinical Trial") AND (checkpoint-inhibitor* OR checkpoint-block* OR checkpoint-therapy OR "immune-checkpoint inhibitor*" OR "immune-checkpoint block*" OR "immunecheckpoint inhibitor*" OR "immunecheckpoint block*" OR Nivolumab OR Opdivo OR ONO-4538 OR ONO4538 OR MDX-1106 OR MDX1106 OR BMS-936558 OR BMS936558 OR Pembrolizumab OR SCH-900475 OR SCH900475 OR Keytruda OR MK-3475 OR MK3475 OR Lambrolizumab OR Cemiplimab OR REGN2810 OR Toripalimab OR Sinitilimanb OR Camrelizumab OR SHR-1210 OR SHR1210 OR Tislelizumab OR Pidilizumab OR CT-011 OR AMP-514 OR MEDI0680 OR AMP-224 OR PDR001 OR Atezolizumab OR MSB0010718C OR RO5541267 OR MPDL-3280A OR MPDL3280A OR Tecentriq OR RG-7446 OR RG7446 OR Durvalumab OR MEDI-4736 OR MEDI4736 OR Imfinzi OR Avelumab OR MSB-0010682 OR MSB0010682 OR Bavencio OR MSB-0010718C OR MSB0010718C OR BMS-936559 OR BMS936559 OR MDX-1105 OR MDX1105 OR anti-PD-1 OR anti-PD-L1 OR anti-PDL1 OR anti-PDL-1 OR anti-PD1 OR ("Programmed Cell Death-1" OR "Programmed Cell Death Ligand-1" OR "Programmed Cell Death-Ligand 1" OR "Programmed Death-Ligand 1" OR "Programmed Death Ligand-1" OR "Programmed Death-1"OR "Programmed Cell Death protein-1" OR PD-1 OR PD-L1 OR PDL-1 OR PDL1 OR PD1 OR PDCD1LG1 OR "programmed cell death 1 ligand 1" OR B7H1 OR B7-H1 OR CD279 OR CD274)))
