# Supplementary material for: Trends in the Research Into Immune Checkpoint Blockade by Anti-PD1/PDL1 Antibodies in Cancer Immunotherapy: A Bibliometric Study
Source: Front Pharmacol. 2021 Aug 17;12:670900. doi: 10.3389/fphar.2021.670900 (PMC8418110; doi:10.3389/fphar.2021.670900)
Supplement: Supplementary file 7 [file Table5.docx]

**Supplementary Table 5. Top 15 documents of PD1/PDL1 molecule sorted by GCS.**

| **Title** | **Source** | **Year** | **GCS** | **GCS per year** |
| --- | --- | --- | --- | --- |
| Safety, activity, and immune correlates of anti-PD-1 antibody in cancer | TOPALIAN SL, N ENGL J MED | 2012 | 6460 | 718 |
| Safety and activity of anti-PD-L1 antibody in patients with advanced cancer | BRAHMER JR, N ENGL J MED | 2012 | 4112 | 457 |
| Nivolumab versus Docetaxel in Advanced Nonsquamous Non-Small-Cell Lung Cancer | BORGHAEI H, N ENGL J MED | 2015 | 3806 | 634 |
| Nivolumab versus Docetaxel in Advanced Squamous-Cell Non-Small-Cell Lung Cancer | BRAHMER J, N ENGL J MED | 2015 | 3739 | 623 |
| Combined Nivolumab and Ipilimumab or Monotherapy in Untreated Melanoma | LARKIN J, N ENGL J MED | 2015 | 3488 | 581 |
| PD-1 Blockade in Tumors with Mismatch-Repair Deficiency | LE DT, N ENGL J MED | 2015 | 3271 | 545 |
| Cancer immunology. Mutational landscape determines sensitivity to PD-1 blockade in non-small cell lung cancer | RIZVI NA, SCIENCE | 2015 | 3183 | 530 |
| Pembrolizumab versus Chemotherapy for PD-L1-Positive Non-Small-Cell Lung Cancer | RECK M, N ENGL J MED | 2016 | 2883 | 577 |
| Nivolumab in previously untreated melanoma without BRAF mutation | ROBERT C, N ENGL J MED-a | 2015 | 2650 | 442 |
| Pembrolizumab for the treatment of non-small-cell lung cancer | GARON EB, N ENGL J MED | 2015 | 2617 | 436 |
| Tumor-associated B7-H1 promotes T-cell apoptosis: a potential mechanism of immune evasion | DONG HD, NAT MED | 2002 | 2586 | 136 |
| Pembrolizumab versus Ipilimumab in Advanced Melanoma | ROBERT C, N ENGL J MED | 2015 | 2520 | 420 |
| Nivolumab plus ipilimumab in advanced melanoma | WOLCHOK JD, N ENGL J MED | 2013 | 2498 | 312 |
| An inhibitor of Bcl-2 family proteins induces regression of solid tumours | OLTERSDORF T, NATURE | 2005 | 2445 | 153 |
| PD-1 blockade induces responses by inhibiting adaptive immune resistance | TUMEH PC, NATURE | 2014 | 2440 | 349 |
