# Supplementary material for: Trends in the Research Into Immune Checkpoint Blockade by Anti-PD1/PDL1 Antibodies in Cancer Immunotherapy: A Bibliometric Study
Source: Front Pharmacol. 2021 Aug 17;12:670900. doi: 10.3389/fphar.2021.670900 (PMC8418110; doi:10.3389/fphar.2021.670900)
Supplement: Supplementary file 8 [file Table3.docx]

**Supplementary Table 3. Top 15 meta-analysis of anti-PD1/PDL1 sorted by GCS.**

| **Title** | **Source** | **Year** | **GCS** | **GCS per year** |
| --- | --- | --- | --- | --- |
| Checkpoint Inhibitors in Metastatic EGFR-Mutated Non-Small Cell Lung Cancer-A Meta-Analysis | LEE CK, J THORAC ONCOL | 2017 | 278 | 69.5 |
| Incidence of Programmed Cell Death 1 Inhibitor-Related Pneumonitis in Patients With Advanced Cancer: A Systematic Review and Meta-analysis | NISHINO M, JAMA ONCOL | 2016 | 185 | 37.0 |
| Fatal Toxic Effects Associated With Immune Checkpoint Inhibitors: A Systematic Review and Meta-analysis | WANG DY, JAMA ONCOL | 2018 | 175 | 58.3 |
| PD-L1 expression in cancer patients receiving anti PD-1/PD-L1 antibodies: A systematic review and meta-analysis | GANDINI S, CRIT REV ONCOL /HEMATOL | 2016 | 160 | 32.0 |
| Incidence of Endocrine Dysfunction Following the Use of Different Immune Checkpoint Inhibitor Regimens: A Systematic Review and Meta-analysis | BARROSO-SOUSA R, JAMA ONCOL | 2018 | 148 | 49.3 |
| Characterisation and management of dermatologic adverse events to agents targeting the PD-1 receptor | BELUM VR, EUR J CANCER | 2016 | 132 | 26.4 |
| Cancer immunotherapy efficacy and patients' sex: a systematic review and meta-analysis | CONFORTI F, LANCET ONCOL | 2018 | 109 | 36.3 |
| Association of body-mass index and outcomes in patients with metastatic melanoma treated with targeted therapy, immunotherapy, or chemotherapy: a retrospective, multicohort analysis | MCQUADE JL, LANCET ONCOL | 2018 | 107 | 35.7 |
| Comprehensive Meta-analysis of Key Immune-Related Adverse Events from CTLA-4 and PD-1/PD-L1 Inhibitors in Cancer Patients | DE VELASCO G, CANCER IMMUNOL RES | 2017 | 106 | 26.5 |
| Incidence of Pneumonitis With Use of Programmed Death 1 and Programmed Death-Ligand 1 Inhibitors in Non-Small Cell Lung Cancer: A Systematic Review and Meta-Analysis of Trials | KHUNGER M, CHEST | 2017 | 105 | 26.2 |
| Clinical and Molecular Characteristics Associated With Survival Among Patients Treated With Checkpoint Inhibitors for Advanced Non-Small Cell Lung Carcinoma: A Systematic Review and Meta-analysis | LEE CK, JAMA ONCOL | 2018 | 99 | 33.0 |
| Immune-related adverse events for anti-PD-1 and anti-PD-L1 drugs: systematic review and meta-analysis | BAXI S, BMJ-BRITISH MEDICAL JOURNAL | 2018 | 99 | 33.0 |
| Comparison of efficacy of immune checkpoint inhibitors (ICIs) between younger and older patients: A systematic review and meta-analysis | NISHIJIMA TF, CANCER TREAT REV | 2016 | 88 | 17.6 |
| Immune-Related Adverse Events Associated with Anti-PD-1/PD-L1 Treatment for Malignancies: A Meta-Analysis | WANG PF, FRONT PHARMACOL | 2017 | 88 | 22.0 |
| The role of PD-L1 expression as a predictive biomarker in advanced non-small-cell lung cancer: a network meta-analysis | AGUIAR PN, IMMUNOTHERAPY-a | 2016 | 75 | 15.0 |
