# Supplementary material for: Trends in the Research Into Immune Checkpoint Blockade by Anti-PD1/PDL1 Antibodies in Cancer Immunotherapy: A Bibliometric Study
Source: Front Pharmacol. 2021 Aug 17;12:670900. doi: 10.3389/fphar.2021.670900 (PMC8418110; doi:10.3389/fphar.2021.670900)
Supplement: Supplementary file 10 [file Table4.docx]

**Supplementary Table 4：Top 15 documents of PD1/PDL1 molecule sorted by LCS.**

| **Title** | **Source** | **Year** | **LCS** | **GCS** |
| --- | --- | --- | --- | --- |
| Predictive correlates of response to the anti-PD-L1 antibody MPDL3280A in cancer patients | HERBST RS, NATURE | 2014 | 1113 | 2288 |
| Tumor-associated B7-H1 promotes T-cell apoptosis: a potential mechanism of immune evasion | DONG HD, NAT MED | 2002 | 1097 | 2586 |
| PD-1 blockade induces responses by inhibiting adaptive immune resistance | TUMEH PC, NATURE | 2014 | 1062 | 2440 |
| Cancer immunology. Mutational landscape determines sensitivity to PD-1 blockade in non-small cell lung cancer | RIZVI NA, SCIENCE | 2015 | 1033 | 3183 |
| Pembrolizumab versus docetaxel for previously treated, PD-L1-positive, advanced non-small-cell lung cancer (KEYNOTE-010): a randomised controlled trial | HERBST RS, LANCET | 2016 | 813 | 2387 |
| Colocalization of inflammatory response with B7-h1 expression in human melanocytic lesions supports an adaptive resistance mechanism of immune escape | TAUBE JM, SCI TRANSL MED | 2012 | 673 | 1226 |
| Association of PD-1, PD-1 ligands, and other features of the tumor immune microenvironment with response to anti-PD-1 therapy | TAUBE JM, CLIN CANCER RES | 2014 | 617 | 1188 |
| Phase I study of single-agent anti-programmed death-1 (MDX-1106) in refractory solid tumors: safety, clinical activity, pharmacodynamics, and immunologic correlates | BRAHMER JR, J CLIN ONCOL | 2010 | 521 | 1644 |
| Immune checkpoint blockade: a common denominator approach to cancer therapy | TOPALIAN SL, CANCER CELL | 2015 | 484 | 1408 |
| MPDL3280A (anti-PD-L1) treatment leads to clinical activity in metastatic bladder cancer | POWLES T, NATURE | 2014 | 463 | 1330 |
| Atezolizumab versus docetaxel in patients with previously treated non-small-cell lung cancer (OAK): a phase 3, open-label, multicentre randomised controlled trial | RITTMEYER A, LANCET | 2017 | 442 | 1420 |
| PD-L2 is a second ligand for PD-1 and inhibits T cell activation | LATCHMAN Y, NAT IMMUNOL | 2001 | 436 | 1580 |
| Atezolizumab versus docetaxel for patients with previously treated non-small-cell lung cancer (POPLAR): a multicentre, open-label, phase 2 randomised controlled trial | FEHRENBACHER L, LANCET | 2016 | 413 | 1145 |
| Atezolizumab in patients with locally advanced and metastatic urothelial carcinoma who have progressed following treatment with platinum-based chemotherapy: a single-arm, multicentre, phase 2 trial | ROSENBERG JE, LANCET | 2016 | 410 | 1441 |
| Survival, durable tumor remission, and long-term safety in patients with advanced melanoma receiving nivolumab | TOPALIAN SL, J CLIN ONCOL | 2014 | 405 | 1299 |
